# Correlating axial and equatorial ligand field effects to the single-molecule magnet performances of a family of dysprosium bis-methanediide complexes†

**DOI:** 10.1039/d1sc00238d

**Published:** 2021-03-02

**Authors:** Lewis R. Thomas-Hargreaves, Marcus J. Giansiracusa, Matthew Gregson, Emanuele Zanda, Felix O'Donnell, Ashley J. Wooles, Nicholas F. Chilton, Stephen T. Liddle

**Affiliations:** Department of Chemistry, The University of Manchester Oxford Road Manchester M13 9PL UK steve.liddle@manchester.ac.uk nicholas.chilton@manchester.ac.uk

## Abstract

Treatment of the new methanediide–methanide complex [Dy(SCS)(SCSH)(THF)] (**1Dy**, SCS = {C(PPh_2_S)_2_}^2−^) with alkali metal alkyls and auxillary ethers produces the bis-methanediide complexes [Dy(SCS)_2_][Dy(SCS)_2_(K(DME)_2_)_2_] (**2Dy**), [Dy(SCS)_2_][Na(DME)_3_] (**3Dy**) and [Dy(SCS)_2_][K(2,2,2-cryptand)] (**4Dy**). For further comparisons, the bis-methanediide complex [Dy(NCN)_2_][K(DB18C6)(THF)(toluene)] (**5Dy**, NCN = {C(PPh_2_NSiMe_3_)_2_}^2−^, DB18C6 = dibenzo-18-crown-6 ether) was prepared. Magnetic susceptibility experiments reveal slow relaxation of the magnetisation for **2Dy–5Dy**, with open magnetic hysteresis up to 14, 12, 15, and 12 K, respectively (∼14 Oe s^−1^). Fitting the alternating current magnetic susceptibility data for **2Dy–5Dy** gives energy barriers to magnetic relaxation (*U*_eff_) of 1069(129)/1160(21), 1015(32), 1109(70), and 757(39) K, respectively, thus **2Dy–4Dy** join a privileged group of SMMs with *U*_eff_ values of ∼1000 K and greater with magnetic hysteresis at temperatures >10 K. These structurally similar Dy-components permit systematic correlation of the effects of axial and equatorial ligand fields on single-molecule magnet performance. For **2Dy–4Dy**, the Dy-components can be grouped into **2Dy–cation**/**4Dy** and **2Dy–anion**/**3Dy**, where the former have almost linear C=Dy=C units with short average Dy=C distances, and the latter have more bent C=Dy=C units with longer average Dy=C bonds. Both *U*_eff_ and hysteresis temperature are superior for the former pair compared to the latter pair as predicted, supporting the hypothesis that a more linear axial ligand field with shorter M–L distances produces enhanced SMM properties. Comparison with **5Dy** demonstrates unusually clear-cut examples of: (i) weakening the equatorial ligand field results in enhancement of the SMM performance of a monometallic system; (ii) a positive correlation between *U*_eff_ barrier and axial linearity in structurally comparable systems.

## Abstract 2

Studies on equatorial donor and C=Dy=C angle variation effects on energy barriers to the slow relaxation of magnetisation are reported.

## Graphical abstract

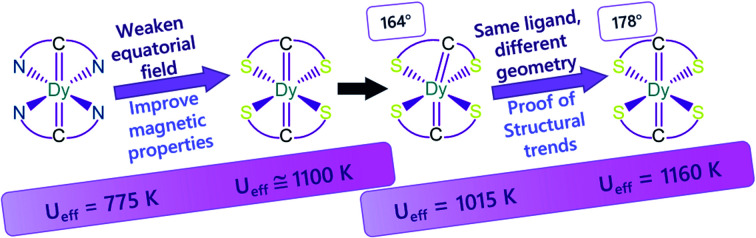

## Introduction

Lanthanide (Ln) Single Molecule Magnets (SMMs) are of burgeoning interest due to their potential applications in high density storage and quantum computing.^1^ Following the discovery that single Ln-ions can function as effective SMMs,^2^ there has been a huge development in the field.^3^ Ln-based SMMs have been amenable to systematic improvement by optimisation of the crystal field (CF) generated by the coordination environment in order to best stabilise the most magnetic projections of the spin–orbit coupled total angular momentum (*m*_*J*_ states).^4^ This approach has permitted design of large barriers to magnetisation reversal, *U*_eff_, over which magnetic relaxation occurs with an Arrhenius-like exponential temperature dependence, and thus larger *U*_eff_ values should lead to slower magnetic relaxation at a given temperature. This is well established for Dy^III^, where near-linear coordination environments stabilise the *m*_*J*_ = |±15/2〉 ground state.^1*b*,3*c*,5^ The preparation and computational investigation of prepared compounds has been instrumental in reinforcing and developing the theory behind SMMs, and yet very few studies have explicitly probed the correlation between axial and equatorial CF effects.^5*g*^

The systematic effect of equatorial donors has been shown in polymetallic cyclopentadienyl systems by descending the group 15/16 elements,^6^ but the only monometallic examples are that of substitution of a chloride for a bromide in [Dy(bbpen)X] (where X = Cl, Br; H_2_bbpen = *N*,*N*-bis(2-hydroxybenzyl)-*N*,*N*-bis(2-methylpyridyl)ethylenediamine) and [Dy(Mes*O)_2_(THF)_2_X] (where X = Cl, Br and I and Mes* = 2,4,6-tri-*tert*-butylphenyl).^3*b*,3*l*^ There have also been further investigations into the effect of ligand properties on magnetic performance^7^ with *Pc* ((C_6_H_4_C_2_N)_4_N_4_),^4*c*^ Cp^R^ systems,^8^ and a recent extensive study of axiality in pentagonal bipyramidal alkoxide SMMs, as particular highlights.^9^ Each of these studies demonstrated the effect of increased axial donor strength on magnetic properties, however none have specifically correlated the effect of axial linearity within a series of comparable systems. Furthermore, a recent study demonstrated that equatorial sulfur donors enabled *U*_eff_ barriers as high as 638 K, with computational investigations showing that heavier group 16 elements would likely further increase the barrier.^10^ To date, the effect of axial linearity on magnetic performance has only ever been modelled computationally or observed as a general qualitative trend for incomparable systems.^4*e*^

A recent breakthrough has been the advent of dysprosocenium cations [Dy(Cp^R^)(Cp^R’^)]^+^ (Cp = cyclopentadienyl), which have *U*_eff_ values ranging from 1760 to 2217 K and record zero field cooled (ZFC)/field cooled (FC) (*T*_B1_), hysteresis measurement (*T*_H_), and 100 second relaxation (*T*_B2_) blocking temperatures of 52, 80, and 67 K, respectively.^3*d*,8,11^ The vastly improved SMM properties of the dysprosocenium cations are thought to be due to the constrained vibrational modes of the five-membered Cp rings,^3*d*,3*j*,8,12^ suggesting more rigid ligand environments are beneficial. Indeed, some of us have recently suggested that quantum tunnelling of the magnetisation (QTM), which is responsible for fast relaxation at zero field and has been a blight on Ln-based SMMs, could be enhanced by flexible ligand environments, and thus ligand rigidity seems key to improving performance.^3*c*,3*d*,12*c*,13^

There are now an increasing number of Ln SMMs in the literature with *U*_eff_ barriers over 1000 K ^3*b*–*d*,5*d*,8,9,11,14^ but few systematic magneto-structural studies of coordination geometry.^3*l*,8^ Whilst this suggests that the current level of understanding of SMM behaviour is effective, methodical testing and evaluation of coordination geometry is required to develop the properties of SMMs to be functional at practical temperatures. This is particularly important for compounds that are not part of the successful families of dysprosocenium cations or pentagonal bipyramidal SMMs if the scope of the field is to be systematically expanded.

We have been attempting to prepare Dy^III^ SMMs that feature large *U*_eff_ barriers as well as rigid multi-dentate ligands. In previous work we constructed an SMM with *trans*-methanediide (formally C^2−^) donors supported with neutral imido donors in the equatorial plane, *viz.* [Dy(NCN)_2_][K(18C6)(THF)_2_] (**a-Dy**) (NCN = {C(PPh_2_NSiMe_3_)_2_}^2−^, 18C6 = 18-crown-6 ether), which has *U*_eff_ = 721 and 813 K with *T*_B1_/*T*_H_ = 10 K (*T*_B2_ not measured).^5*c*^ In order to improve this system, we proposed analogues of NCN where the equatorial imido donors were replaced with softer sulfur donors, therefore reducing the strength of the equatorial interaction. Here, we report a structurally similar ligand SCS = {C(PPh_2_=S)_2_}^2−^ where the NSiMe_3_ groups have been replaced with softer S donors, and this family of molecules permits a systematic magneto-structural investigation and correlation of axial/linear and equatorial ligand field effects on the SMM performances of these complexes. Our combined experimental and computational investigation reveals that this replacement increases *U*_eff_ on the order of 40%, and increases to the *T*_B_ behaviour by several K. Furthermore, a positive correlation between linearity and *U*_eff_ barrier is specifically demonstrated here for the first time within comparable systems.

## Results

### Synthesis

**Scheme 1:**
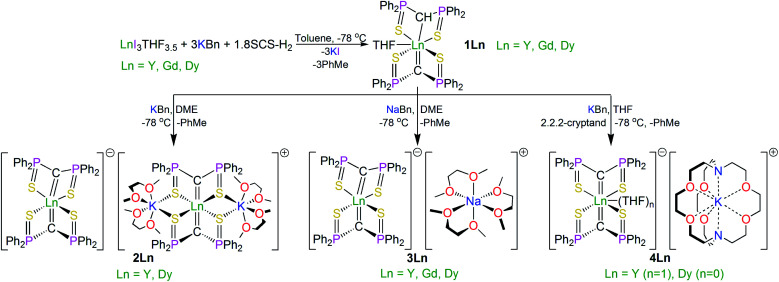
Synthetic routes to the bis-methanediide complexes **1Ln–4Ln**, Ln = Dy, Y, Gd. The synthesis of **5Dy** is very similar, utilising H_2_C(PPh_2_NSiMe_3_)_2_ instead of H_2_C(PPh_2_S)_2_ and dibenzo-18-crown-6 ether as the auxiliary ligand.

The SCS ligand has been used extensively throughout the d- and f-blocks, including previous reports of bis-SCS Ln complexes of Sm and Tm.^15^ Those compounds were prepared by reaction of SCS–Li_2_ with LnI_3_THF_3.5_ (Ln = Sm, Tm) *via* salt elimination. However, in order to introduce more modular variation of the alkali metal, since this was anticipated to provide greater opportunities for the structural variations needed to underpin a magneto-structural correlation study, we adapted the alkane elimination route previously used in our NCN work to the synthesis of the bis-SCS Ln complexes reported here, Scheme 1.^5*c*^ Accordingly, treatment of LnI_3_THF_3.5_ with three equivalents of KCH_2_Ph and a sub-stoichiometric amount of SCSH_2_ produced the heteroleptic methanediide-methanide complex [Dy(SCS)(SCSH)(THF)] (**1Dy**), which was isolated as colourless crystals in 90% yield with toluene as a byproduct. A sub-stoichiometric amount of SCSH_2_ ensures the clean isolation of pure **1Dy** since any unreacted SCSH_2_ has a very similar solubility to **1Dy** and is thus impracticable to separate during work-up.

With **1Dy** secured, the target bis-methanediide derivatives were prepared by deprotonation with MCH_2_Ph reagents (M = Na, K) in the presence of auxiliary ethers (DME or 2,2,2-cryptand), yielding colourless crystalline [Dy(SCS)_2_][Dy(SCS)_2_(K(DME)_2_)_2_] (**2Dy**), [Dy(SCS)_2_][Na(DME)_3_] (**3Dy**) and [Dy(SCS)_2_][K(2,2,2-cryptand)] (**4Dy**) in isolated crystalline yields of 81, 73, and 72%, respectively.

For completeness and to aid characterisation, we prepared diamagnetic **1Y–4Y** which are largely isostructural to their Dy-analogues except for **4Y** which contains a molecule of coordinated THF that is not present in **4Dy**. The ^31^P NMR spectra are particularly diagnostic in these systems, with methanide and methanediide resonances observed for **1Y** at ∼33 and ∼14 ppm, respectively, whilst compounds **2Y**, **3Y**, and **4Y** all demonstrate a single methanediide resonance at ∼14 ppm indicating equivalent SCS ligands in solution on the NMR timescale. The magnetic data of **2Dy–4Dy** are modelled well by the *ab initio* calculations (*vide infra*), suggesting minimal influence of intermolecular forces on the magnetic behaviour of these complexes and thus dilution studies were not required. Complexes **1Y–4Y** are therefore not discussed any further but details are included in the ESI for completeness.

Since Dy is not amenable to electron paramagnetic resonance (EPR) studies, we prepared **3Gd** from **1Gd** so that the isotropic Gd ion in **3Gd** could be probed to determine its crystal field parameters spectroscopically as a proxy to the Dy-congeners.

Lastly, for further comparisons, the bis-methanediide complex [Dy(NCN)_2_][K(DB18C6)(THF)(toluene)] (**5Dy**, NCN = {C(PPh_2_NSiMe_3_)_2_}^2−^, DB18C6 = dibenzo-18-crown-6 ether) was prepared; its synthesis largely followed the same strategy as the preparations of **3Dy**, **4Dy**, and **a-Dy** and is unremarkable.

### Solid state structures

**Fig. 1:**
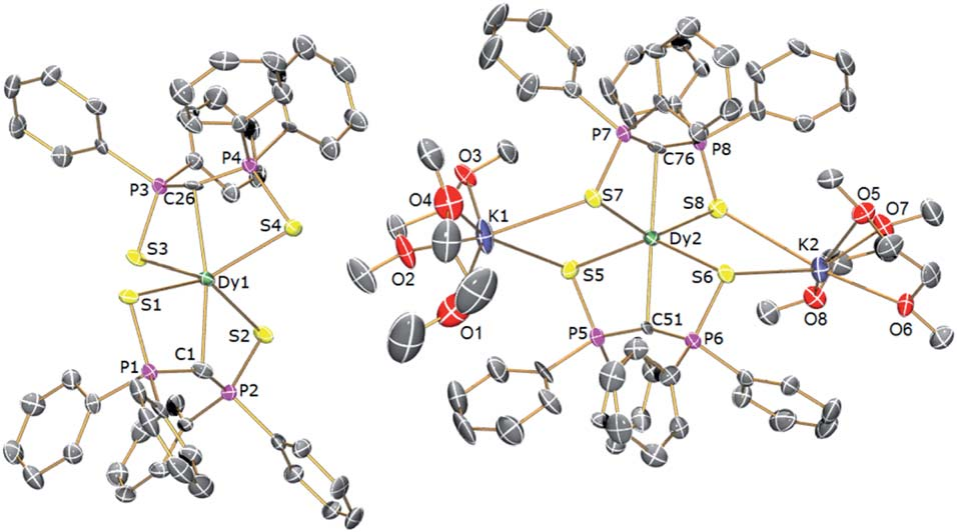
Solid-state structures of **2Dy** at 150 K with selective labelling and displacement ellipsoids set at 40% probability. Hydrogen atoms and minor disorder components are omitted for clarity.

**Fig. 2:**
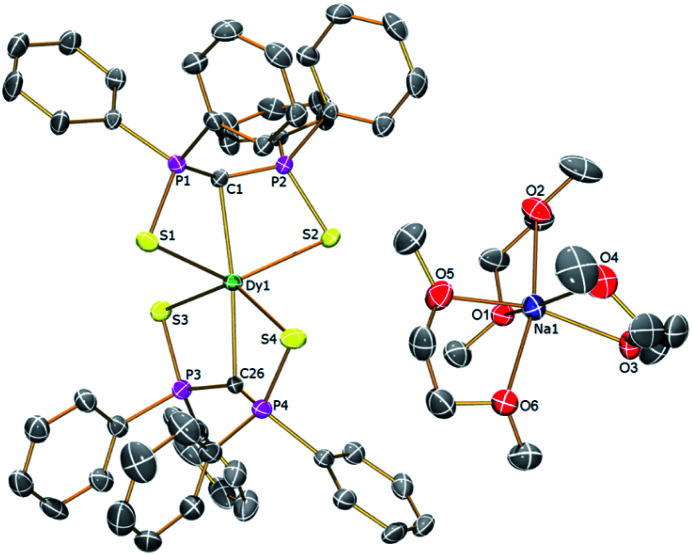
Solid-state structures of **3Dy** at 150 K with selective labelling and displacement ellipsoids set at 40% probability. Hydrogen atoms and minor disorder components are omitted for clarity.

**Fig. 3:**
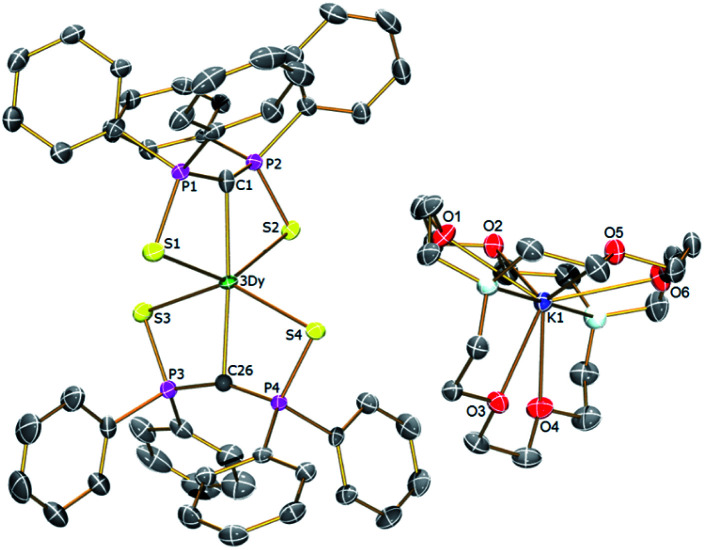
Solid-state structures of **4Dy** at 150 K with selective labelling and displacement ellipsoids set at 40% probability. Hydrogen atoms and minor disorder components are omitted for clarity.

In order to verify the formulations of the complexes reported here we determined their solid-state structures by X-ray diffraction; the structures of **2Dy–4Dy** are shown in Fig. 1–3 and Table 1 and details of the structural determinations of **1Dy**, **3Gd**, **5Dy**, and **1Y–4Y** can be found in the ESI. Compounds **3Dy** and **4Dy** comprise 6-coordinate C_2_S_4_-coordinated Dy complexes as discrete anions, while **2Dy** exhibits two discrete Dy-components as a separated ion pair (hereafter referred to as **2Dy–anion** and **2Dy–cation**). The 7-coordinate **1Dy** precursor has an additional THF coordinated to Dy, and so the resulting C_2_S_4_O-coordination diverges from the axial, linear coordination environment that is sought. For **5Dy**, the steric bulk of SiMe_3_ groups on the NCN ligand prevents coordination of group 1 metals and orthogonally locks the two methanediides to give a C_2_N_4_-coordinated Dy ion. The main difference of **5Dy** compared to **a-Dy** is the K-crown component, but as will be seen these two complexes are magnetically different.

**Table 1:** Comparative structural features of compounds **2Dy–5Dy**

	**2Dy–anion**	**2Dy–cation**	**3Dy**	**4Dy**	**5Dy**
C=Dy=C/°	166.1(3)	178.6(2)	164.01(11)	176.03(11)	176.45(9)
C=Dy/Å	2.432(7), 2.409(8)	2.415(7), 2.390(7)	2.449(3), 2.407(3)	2.381(4), 2.387(3)	2.434(6), 2.431(6)

Typically, bis-SCS Ln complexes exhibit C=Ln=C angles of ∼166°; which is most likely due to only partial transfer of electron density from the methanediide centre to the Ln ion, which tends to produce trigonal pyramidalised carbon centres rather than trigonal planar ones.^15^ This is clearly observed in **3Dy** which shows different C=Dy bond lengths of 2.407(3) and 2.449(3) Å, with the latter tending towards a methanide geometry around the carbon centre. This is also demonstrated by the greater Dy–S–C–S torsion angle of 23° for the longer C=Dy bond in comparison to 16° for the shorter bond, with the same effect observed for **2Dy–anion**. In contrast, the **2Dy–cation** and **4Dy** both have near linear C=Dy=C angles of 178.6(2)° and 176.03(11)°, respectively. In the case of **2Dy–cation**, this is explained by the coordination of K ions to a S atom from each of the Dy(SCS)_2_ moieties, which locks the two SCS ligands into an orthogonal arrangement. A similar effect from the SiMe_3_ groups is observed in **5Dy** (C=Dy=C angle 176.45(9)°), which sterically locks the BIPM ligands orthogonally to each other. However, **4Dy** also displays a large C=Dy=C angle (176.03(11)°), despite the apparent lack of K-coordination or interlocking, and this is most likely due to crystal packing effects fortuitously producing the desired geometry. As expected, the C–Dy=C angle in 7-coordinate **1Dy** is far from linear at 142.76(15)° due to the additional coordinating THF.

The **1Dy** Dy–C and Dy=C bond lengths display a clear distinction between the methanide (2.757(4) Å) and methanediide distances (2.326(5) Å). The SCS ligand is able to form a short C=Dy bond in this case due to the elongation of the weaker methanide ligand *trans* to it, which reduces steric repulsion between the ligands and allows the methanediide ligand to approach closer to the metal. Comparing the Dy=C bond lengths of **2Dy–cation** (2.391(7) and 2.415(7) Å) and **2Dy–anion** (2.409(8) and 2.432(7) Å) we find that they are statistically indistinguishable, despite the difference in C=Dy=C bond angle. The Dy=C bond lengths of **3Dy** are comparable to that of **2Dy–anion**, however, possibly due to a further 2° deviation from linearity, a longer Dy=C bond is now statistically distinguishable (2.449(3) Å *vs.* 2.407(3) Å). The Dy=C bond lengths in **4Dy** (2.381(4) and 2.387(3) Å) are remarkably symmetric in comparison to the structures in this series, and are shorter on average than those in **3Dy**, but statistically indistinguishable to those in **2Dy–cation**.

For **5Dy**, the C=Dy bond lengths are significantly longer than those in **4Dy**, both of which have a C=Dy=C angle of around 176°. This can be attributed to a result of the steric bulk of the NCN ligand compared to SCS, which can be intuitively recognised as preventing ligands *trans* to one another from forming closer contacts with the metal centre. However, shorter Dy=C distances in **4Dy** compared to **5Dy** could also be due to reduced donation of electron density from S to Dy in **4Dy** compared to N to Dy donation in **5Dy**. All Dy–S bond lengths are within the range of 2.74–2.87 Å and whilst those of **1Dy** are longer on average, there is otherwise no clear trend. As expected, the Dy–N bonds in **5Dy** are much shorter at 2.462(2)–2.516(2) Å.

### EPR spectroscopic characterisation

**Fig. 4:**
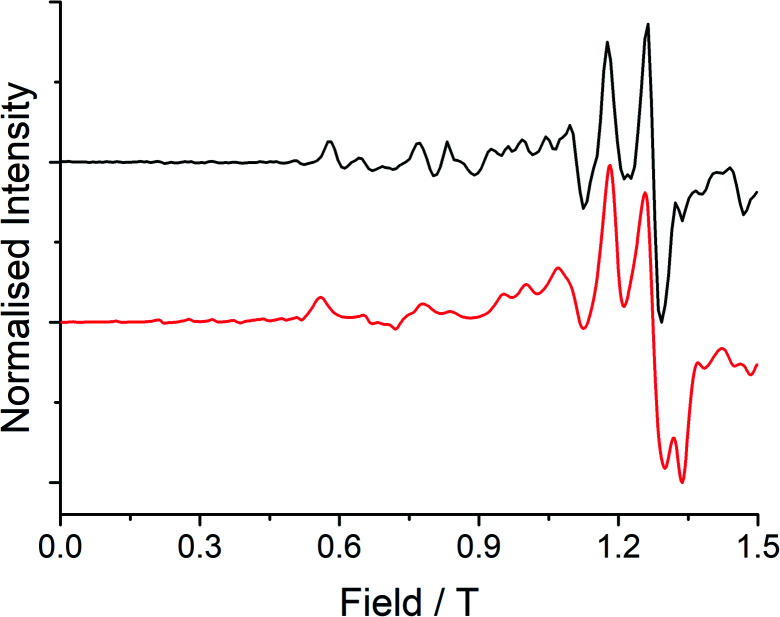
Q-band (33.95491 GHz) EPR spectrum (black line) with simulation (red line) at 5 K of a powdered sample of **3Gd** restrained in eicosane.

Low temperature EPR spectroscopy is an excellent probe of paramagnetic ground states and local symmetry resulting from CF effects. Unfortunately, transitions involving the well-isolated ground *m*_*J*_ = |±15/2〉 Kramers doublet of high-performance Dy SMMs are not possible, and therefore we examined isostructural **3Gd** to probe the axiality of the CF. The EPR spectrum at *Q*-band frequency (33.95491 GHz) for **3Gd** at 5 K, Fig. 4, gives a highly featured spectrum that was modelled using Hamiltonian eqn (1) in PHI^16*a*^ with *g* = 1.983, |*D*| = 0.11 cm^−1^, and |*E*| = 0.0085 cm^−1^ (|*E*/*D*| = 0.08). The sign of the parameters is not obvious from simulation of the spectrum and thus only the magnitudes are reported here.1
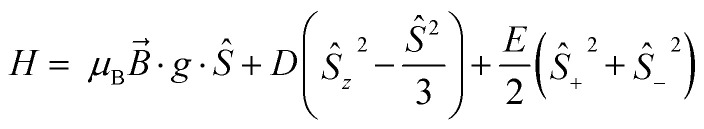


Observation of a large zero-field splitting (ZFS) for **3Gd** is unsurprising given the strongly anisotropic electronic structure of **3Dy** (*vide infra*), however *D* is somewhat smaller and |*E*/*D*| is somewhat larger (*i.e.* more rhombic) than for [Gd(Cp^ttt^)_2_][B(C_6_F_5_)_4_], which has |*D*| = 0.3347 cm^−1^ and |*E*| = 0.01629 cm^−1^ (|*E*/*D*| = 0.05).^12*c*^ This corresponds well with the magnetic results obtained here for **3Dy** that show a smaller *U*_eff_ than for [Dy(Cp^ttt^)_2_][B(C_6_F_5_)_4_] (*vide infra*).^3*d*^

### Magnetometry

**Fig. 5:**
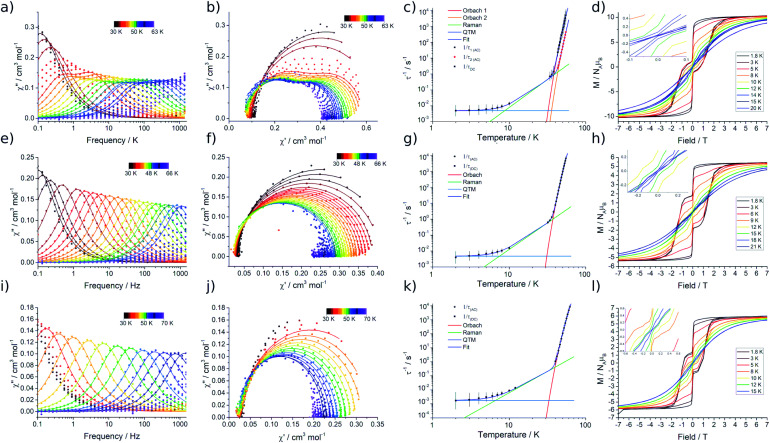
Alternating-current susceptibility (with generalised Debye model fits), Cole–Cole data (data points as coloured dots and fitted curves as lines, same Debye model fits), fitted relaxation data, and magnetic hysteresis measurements (sweep rate of ∼14 Oe s^−1^, insets show zoom-ins at zero field) for **2Dy–4Dy**. (a) **2Dy** 30–63 K (34–38, 43–55 K), (b) **2Dy** from 30–63 K, (c) **2Dy**, (d) **2Dy** 1.8–20 K, (e) **3Dy** 30–66 K (32–55 K), (f) **3Dy** 30–66 K, (g) **3Dy**, (h) **3Dy** 1.8–15 K, (i) **4Dy** 30–70 K (40–62 K), (j) **4Dy** 30–70 K, (k) **4Dy**, (l) **4Dy** 1.8–21 K.

Variable temperature magnetic susceptibility measurements performed on **2Dy** approach the room temperature *χ*_M_*T* value predicted for two non-interacting Dy^III^ ions (28.3 cm^3^ mol^−1^ K), while the data at room temperature for **3Dy–5Dy** are consistent with a single Dy^III^ ion (Fig. S15†). In all cases a subtle decrease in *χ*_M_*T* with reducing temperature is observed, arising from the depopulation of excited CF states, and an abrupt drop at the lowest temperatures is due to magnetic blocking. Alternating Current (AC) susceptibility measurements^16*b*^ reveal frequency dependent peaks in the out-of-phase component (*χ*′′) for **2Dy–5Dy**, Fig. 5a, e, i, and Fig. S16–S19,† indicating slow relaxation of the magnetisation, thus classifying all four compounds as SMMs.

Compound **2Dy** displays irregular-shaped peaks in the out-of-phase AC susceptibility, which clearly resolves into two peaks in a Cole–Cole plot at higher temperatures (Fig. 5b, Tables S3 and S4†). Fitting the AC data with the generalised Debye model reveals two distinct relaxation processes above 43 K and a single relaxation process between 34 and 38 K. For both **3Dy** and **4Dy**, Fig. 5f and j, only one peak in the out-of-phase AC susceptibility is observed (Tables S6 and S7†). Interestingly, the behaviour for **5Dy** is different to its isomer **a-Dy**; multiple relaxation pathways were observed for **a-Dy**, and yet only a single relaxation process is observed for **5Dy** (Fig. S20, Table S10†).^5*c*^

In order to probe magnetic relaxation rates at lower temperatures, we also performed direct current (DC) magnetisation decay measurements in zero field. In all cases we find near mono-exponential decay at higher temperatures (>6 K) and a trend towards slightly multi-exponential decay (stretch parameter *ca.* 0.7) at 2 K (Fig. S21–S24; Tables S5, S8, S9 and S11†). The temperature dependence of the magnetic relaxation rates for **2Dy–5Dy** all display three characteristic regimes; at high temperatures there is an exponential (and for **2Dy** there are two) relaxation process, at intermediate temperatures there is a power-law process, and at the lowest temperatures there is a near temperature independent process. We assign these three processes to Orbach, Raman and QTM mechanisms, respectively, and the data were fitted with eqn (2) and (3); here we convert the distribution (*α*) and stretch (*β*) parameters from the generalised Debye model into estimated standard deviations (esds).^17^ We suggest that the two distinct exponential regions for **2Dy** owe to each of the discrete molecules within the structure, **2Dy–anion** and **2Dy–cation**, as seen in other multimetallic Dy SMMs.^18^ Subsequently, we fit the data using two exponential terms, eqn (3). This contrasts to our previous report on **a-Dy** where two relaxation processes were observed to originate from the single unique Dy^III^ centre as a result of two distinct relaxation pathways involving the 3^rd^ and 4^th^ excited states.^5*c*^ Fitting these data, Fig. 5c, g, and k, gives the parameters in Table 2. All *U*_eff_ values for the SCS SMMs are around ∼1000 K, and that for **5Dy** is substantially lower at ∼750 K; we note that this value is very close the average of the two *U*_eff_ values found previously for **a-Dy** (av. *U*_eff_ = 767 K).2
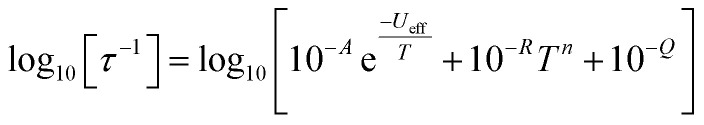
3
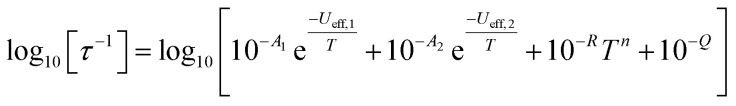


**Table 2:** Comparative magnetic parameters of compounds **2Dy–5Dy**^a^

Sample	*U*_eff_ (K)	*τ*_0_ (s)	*C* (s^−1^ K^−*n*^)	*n*	*τ*_QTM_ (s)	*T*_H_ (K)	*T*_B1_ (K)	*T*_B2_ (K)
**2Dy** (**1**)	1160(21)	10^–11.79(18)^	10^–5.56(69)^	3.50(55)	10^2.29(16)^	14	12	8
**2Dy** (**2**)	1069(129)	10^–12.1(12)^						
**3Dy**	1015(32)	10^–11.82(29)^	10^–5.53(46)^	3.54(32)	10^2.26(14)^	12	11	8
**4Dy**	1109(70)	10^–11.69(56)^	10^–5.39(93)^	3.20(84)	10^2.65(25)^	15	13	12
**5Dy**	757(39)	10^–11.53(45)^	10^–6.22(24)^	4.49(22)	10^1.90(4)^	12	10	—
**a-Dy** (**1**)	721(1)	1.11(3)	3.01(7) × 10^–11^	8	—	10	10	—
**a-Dy** (**2**)	813(1)	0.565(20)	3.55(10) × 10^−9^	6	—			

a The fitting of the relaxation data using eqn (2) and (3) gives the relationships *τ*_0_ = 10^A^ (s), C = 10^−R^ (s^−1^ K^−n^), and *τ*_QTM_ = 10^Q^ (s), where errors are reported in the exponents.

In order to define the blocking temperatures of these molecules, we performed *T*_B1_, *T*_H_, and *T*_B2_ measurements, Fig. 5d, h and l. Owing to the samples being moisture sensitive, the magnetic measurements are performed on samples sealed in borosilicate NMR tubes. In **2Dy**, bifurcation is observed in ZFC/FC susceptibility below *T*_IRREV_ = 30 K, indicating out-of-equilibrium behaviour, with a peak in the ZFC measurement observed at *T*_B1_ = 12 K (Fig. S25†). This large *T*_IRREV_ may be an artefact of delayed temperature equilibration at the sample: for the field-cooling measurement, this would result in a higher true temperature and therefore lower signal than the equilibrium *χ* value when collecting this cooling cycle (and *vice versa*). Therefore, ZFC/FC measurements are also reported with a slower sweep rate allowing longer temperature equilibration between each measurement, subsequently shifting the peak position to lower temperature due to the longer waiting times *T*_B1-slow_ = 8 K (Fig. S25,† ∼0.38 K min^−1^ (fast) and ∼0.031 K min^−1^ (slow)). For **2Dy**, a relaxation rate of 100 seconds is found at *T*_B2_ = 8 K. Magnetic hysteresis loops collected with a sweep rate of *ca.* 15 Oe s^−1^ are open below *T*_H_ = 14 K (Fig. 5d).

ZFC/FC measurements for **3Dy** and **4Dy** show separation at *T*_IRREV_ = 12 and 15 K, respectively. Peaks are present in the ZFC measurements at *T*_B1_ = 11 and 13 K (Fig. S26 and S27,†*T*_B1-slow_ = 7.5 and 9 K with the slower sweep rate), hysteresis loops are open to *T*_H_ = 12 and 15 K (sweep rate ∼14 Oe s^−1^) and *T*_B2_ = 8 and 12 K for **3Dy** and **4Dy**, respectively. For **5Dy** we find ZFC/FC separation below 13 K with a peak in the ZFC measurement at *T*_B1_ = 10 K (*T*_B1-slow_ = 7.5 K) (Fig. S28†), and open hysteresis loops below 12 K (sweep rate ∼14 Oe s^−1^, Fig. S29†). As this molecule has a *τ*_QTM_ value of 79 s, a 100 s blocking temperature cannot be defined. For all hysteresis measurements we observe a step at zero field indicating fast relaxation due to QTM.

### *Ab initio* calculations

**Fig. 6:**
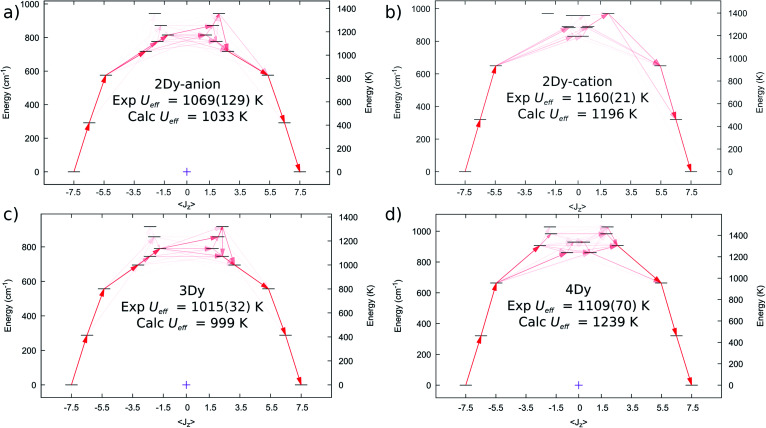
Computed *ab initio* crystal field diagrams for **2Dy–4Dy**. (a) **2Dy–anion**. (b) **2Dy–cation**. (c) **3Dy**. (d) **4Dy**.

We performed complete active space self-consistent field spin–orbit (CASSCF-SO) calculations for **2Dy–5Dy**, employing the single crystal XRD atomic coordinates in all cases (see Methods), Fig. 6. A similar CF-splitting of the ground ^6^H_15/2_ multiplet is observed for the anionic and cationic molecules in **2Dy**, which were performed as separate calculations. The [Dy(SCS)_2_K_2_(DME)_4_]^+^ cation has a slightly greater CF splitting compared to the [Dy(SCS)_2_]^−^ anion (Tables S12 and S13†). In both cases we observe a *m*_*J*_ = |±15/2〉 ground state with principal axis directed towards one of the carbene bonds (deviation in angle to the equivalent carbene bonds with ∠*g*_*z*_-Dy=C76 is 0.552° for **2Dy–cation** and ∠*g*_*z*_-Dy=C1 is 1.832° for **2Dy–anion**, Table S19†). The first two excited states are also highly axial, while the 3^rd^ excited state is highly mixed. Examination of the excited Kramers doublets and the average cartesian magnetic moment transition probabilities^17^ between all states reveals that relaxation *via* the Orbach mechanism would most likely occur through the 3^rd^ excited state in both cases, which are calculated at 1196 and 1033 K, for [Dy(SCS)_2_K_2_(DME)_4_]^+^ and [Dy(SCS)_2_]^−^, respectively. These energies are in excellent agreement with the *U*_eff_ values determined from AC susceptibility for **2Dy** (Fig. 3), and suggest that *U*_eff,1_ = 1160(21) K corresponds to the [Dy(SCS)_2_K_2_(DME)_4_]^+^ cation while *U*_eff,2_ = 1069(129) K corresponds to [Dy(SCS)_2_]^−^; note the midpoint of the experimental parameters is in good agreement, despite the large uncertainty due to the significant esds in the AC data.

The electronic structure of **3Dy** similarly shows strong stabilisation of the large *m*_*J*_ projections of the Dy^III^ ion (Fig. 6c, Table S14†), and suggests that Orbach relaxation is most likely to occur *via* the 3^rd^ or 4^th^ excited states (999 K or 1072 K, Fig. 6c), which is in good agreement with the experimental value of *U*_eff_ = 1015(27) K. Meanwhile, CASSCF-SO calculations suggest that **4Dy** has the largest CF splitting of all the SCS analogues, with the 3^rd^ excited state (where relaxation *via* the Orbach mechanism is favoured, Fig. 6d) predicted at 1239 K (Table S15†), higher than the experimental *U*_eff_ = 1109(70) K.

Calculations for **5Dy** show almost pure *m*_*J*_ states for the first three Kramers doublets with a highly mixed 3^rd^ excited doublet at 693 K (Table S16†). However, inspection of the average Cartesian magnetic moment transition probabilities (Fig. S31†) suggests that there is a favourable Orbach relaxation pathway *via* the 5^th^ excited doublet, with 70%|±9/2〉 at 838 K, having the highest transition probability out of the 2^nd^ excited doublet with 96%|±11/2〉. This would suggest a larger barrier than observed experimentally (*U*_eff_ = 757(39) K), however there is likely relaxation *via* the 3^rd^ and 4^th^ excited doublets at 693 and 793 K; indeed, the average energy of these three doublets is 774 K.

CASSCF-SO calculations of **3Gd** predict the ZFS parameters of the system (Table S18†). Recall we were unable to determine the sign of *D* or *E* from the spectra, however CASSCF-SO predicts *D* = −0.078 and *E* = −0.010 cm^−1^, which are close to the experimental magnitudes of |*D*| = 0.11 cm^−1^ and |*E*| = 0.0085 cm^−1^, and thus we suggest both *D* < 0 and *E* < 0 here.

## Discussion

The local coordination environment is responsible for the CF splitting of the *J* = 15/2 multiplet of Dy^III^ and the origin of the *U*_eff_ energy barrier to magnetic relaxation. We have previously shown that classical electrostatics dominate the magnetic anisotropy for Dy^III^ complexes,^4*d*^ and thus simple parameters such as bond lengths and angles for the charged donor atoms have a significant impact on the *U*_eff_ barrier. Due to the dominant influence of the two near-linear *trans*-disposed C^2−^ donor atoms of the bis-methanediide motif, the lowest three Kramers doublets for **2Dy–5Dy** are almost pure |±15/2〉, |±13/2〉, and |±11/2〉 states quantised along the principal axes of the ground state doublets (the deviation angle of these axes are given in Table S19†), and the third excited state is highly mixed in all cases. The geometrical differences between the structures seem to have a small influence on the composition of these states, although, the main effect is changes in their energies (Tables S12–S16†).

**Fig. 7:**
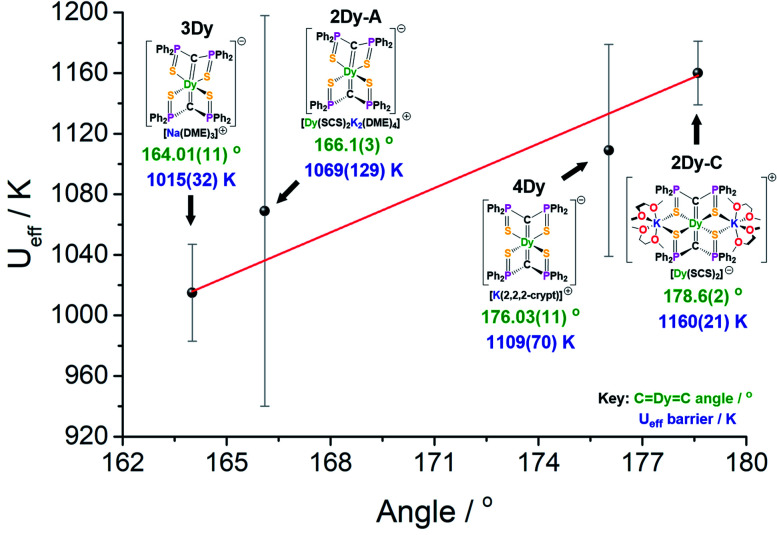
*U*_eff_ barrier as a function of C=Dy=C angle for **2Dy–4Dy**.

The four SCS complexes can be grouped into two pairs: the first consisting of **2Dy–cation** and **4Dy**, which have the largest C=Dy=C angles (∼176–179°) and the shorter av. Dy=C bonds (2.38–2.40 Å), and the second consisting of **2Dy–anion** and **3Dy** that have the smaller C=Dy=C angles (∼164–166°) and longer av. Dy=C bonds (2.42–2.43 Å), Table 1. Based on simple electrostatics, the samples with the most linear arrangement of the C=Dy=C motif and the shortest Dy=C bonds would be expected to have the highest *U*_eff_ value. Therefore, we would expect the **2Dy–cation** and **4Dy** to have larger *U*_eff_ than the **2Dy–anion** and **3Dy**, and this is found to be exactly the case experimentally (Fig. 7). Furthermore, by replacing the hard, equatorial N-donors with softer S-donors, the *U*_eff_ values for all of **2Dy–4Dy** are larger than **5Dy** by approximately 40%. This effect is most clearly demonstrated by comparison of **4Dy** to **5Dy**, which both have C=Dy=C angles of approximately 176° and are free of alkali-metal coordination, with *U*_eff_ barriers of 1109(70) and 757(39) K respectively.

We observe an interesting situation for the orientation of the principal *g*_*z*_ axis of the ground |±15/2〉 state for the present compounds. This axis lies along the average Dy=C vector for **5Dy** (Table S19†), as expected for two strong *trans*-methanediide donors, and yet despite similar variations between the pairs of Dy=C bond lengths in any given complex (*ca.* 0.04 Å), this is not repeated in the SCS variants. For each of the SCS complexes, the principal axis surprisingly aligns with the longer Dy=C bond (Table S19†). This orientation is not reconcilable with simple electrostatic interactions and reveals more complex interactions are at play. Additionally, the **2Dy–cation**, which has the largest *U*_eff_ value and C=Dy=C angle, also has the smallest average ∠*g*_*z*_-Dy=C angles at 0.84 and 0.55°. This is due to the coordination of potassium ions locking the molecule in place and increasing the rigidity of the system, as observed in the Cp-based systems.^3*d*^ Furthermore, despite **4Dy** having the shortest average and most symmetrical Dy=C distances, its *U*_eff_ value is less than that of the **2Dy–cation** (1109 *vs.* 1160 K, respectively). We attribute this to the presence of the potassium ions bound to the sulphur groups in the **2Dy–cation**, whose role could be two-fold: whilst enabling the C=Dy=C bond angle to be more linear (176° *vs.* 179°), they also likely polarise negative charge away from the S-donors, weakening their donor strength to Dy. To examine this latter effect, we have performed a CASSCF-SO calculation on the [Dy(SCS)_2_K_2_(DME)_4_]^+^ structure where the {K(DME)_2_}^+^ moieties were removed (Table S20†). The energy spectrum reveals a slight decrease in CF splitting, reducing the energy of the highly mixed 3^rd^ excited state by about 100 K. It would be interesting to isolate the **2Dy–cation** to measure its properties without the neighbouring anion, however, thus far all experimental attempts have been unsuccessful.

An interesting result of our measurements on **5Dy** reveals a single Orbach relaxation process with *U*_eff_ = 757(39) K, in contrast to the parent isomer **a-Dy** which shows two relaxation pathways with *U*_eff_ = 721(1) and 813(1) K. Comparison of the CASSCF-SO energy spectra of these two species shows only minor differences between the CF states, with the 3^rd^ and 4^th^ excited states for **a-Dy** calculated at 742 and 810 K. There is no clear reason why the relaxation appears as a single mechanism for **5Dy** (*α* < 0.05) and the calculated barrier could be masking multiple pathways as it is unclear which CF states are involved in the Orbach relaxation mechanism. This work clearly shows how the *U*_eff_ energy barrier can be affected based on minor changes to the geometry and electrostatics of the coordination environment.

The comparison of the ZFC/FC measurements performed on these analogues highlights an important issue with the definition of *T*_B1_. As a number of high performing SMMs are temperature and/or moisture sensitive, they require similar preparation to the sealed NMR tubes used for the SCS samples presented here. Therefore, since there is no standard sweep rate for the assignment of *T*_B1_, it is difficult to be sure that reported relaxation behaviour has origins from SMM blocking, or additionally is influenced by temperature equilibration issues at the sample.

As shown in Fig. 7, there is a positive correlation between the *U*_eff_ barrier and C=Dy=C angle. A similar trend can be shown for average ∠*g*_*z*_-Dy=C angle against *U*_eff_ barrier which, as expected, demonstrates a negative correlation (Fig. S32†). However, it is important to note some caveats. Firstly, the slight structural deviations between each bis-SCS structure must be addressed. As mentioned, the C=Dy bonds of **4Dy** are marginally shorter than most other C=Dy bond lengths, although upon considering the magnitude of this difference, it is unlikely to have a major effect. Additionally, the coordinated potassium ions in the **2Dy–cation** are expected to remove electron density from the S-atoms and consequently weaken their equatorial presence. Although computational models suggest that without this coordination the *U*_eff_ barrier would decrease, the magnitude of this effect is not great enough to disrupt the trend in linearity. Secondly, there is a gap in the middle region of the graph that is devoid of data points. As there is no clear method for producing bis-methanediide SCS compounds with specific C=Dy=C angles, targeting this gap would present a major challenge. Thirdly, the uncertainty involved in calculation of the *U*_eff_ value is different for each point, and indeed has been correlated to atomic displacement parameters in crystal structures.^19^ The trend line passes directly through the two data points with smallest error. Given the experimental limitations, this data, along with the supported computational validation of *U*_eff_, experimentally demonstrate a magneto-structural trend with respect to linearity with minimal deviations in structural facets, particularly compared to the existing literature.

## Conclusions

We have prepared a series of SMMs with high energy barriers though deliberate tailoring of the coordination environment of an existing SMM in order to weaken the equatorial donors. Replacement of equatorial N-donors for softer S-donors results in a *ca.* 40% increase in *U*_eff_. The longer Dy–S bonds and more defuse electron density reduces the effect of the equatorial CF and increases the stabilisation of the highly magnetic states of Dy^III^, as predicted. Additionally, this results in an increase in the blocking temperature, up to 15 K. The ability to isolate a number of [Dy(SCS)_2_]^+/−^ variants has allowed the observation of a trend in *U*_eff_ across an analogous series, relating to the linearity of the bis-methanediide coordination.

## Author contributions

L. R. T.-H., M. G., E. Z., and F. O’D. prepared and characterised the complexes. L. R. T.-H. and M. J. G. obtained and analysed the magnetic data. A. J. W. collected, solved, and refined the crystal structures. N.F.C. and S.T.L. originated the central idea, directed the research, and analysed all the data. L. R. T.-H., M. J. G., N. F. C., and S. T. L. wrote the manuscript with input from all the other authors.

## Conflicts of interest

There are no conflicts to declare.

## Supplementary Material

SC-012-D1SC00238D-s001

SC-012-D1SC00238D-s002
