# Supplementary material for: Correlating axial and equatorial ligand field effects to the single-molecule magnet performances of a family of dysprosium bis-methanediide complexes
Source: Chem Sci. 2021 Mar 2;12(11):3911–20. doi: 10.1039/d1sc00238d (PMC8179472; doi:10.1039/d1sc00238d)
Supplement: SC-012-D1SC00238D-s001 [file SC-012-D1SC00238D-s001.pdf]

Electronic Supplementary Material (ESI) for Chemical Science.
This journal is © The Royal Society of Chemistry 2021

- Electronic Supplementary Information -

Correlating Axial and Equatorial Ligand Field Effects to the Single-Molecule Magnet Performances of a Family of Dysprosium *Bis*-Methanediide Complexes

Lewis R. Thomas-Hargreaves, Marcus J. Giansiracusa, Matthew Gregson, Emanuele Zanda, Felix O'Donnell, Ashley J. Wooles, Nicholas F. Chilton* and Stephen T. Liddle*

Department of Chemistry, The University of Manchester, Oxford Road, Manchester, M13 9PL, UK.

*E-mail: steve.liddle@manchester.ac.uk; nicholas.chilton@manchester.ac.uk

Contents

Experimental Methods 2

Figures 10

Tables 28

References 47

Experimental Methods

General

All manipulations were carried out under an inert atmosphere of dry nitrogen or argon using standard Schlenk techniques, or an MBraun UniLab glovebox operating under an atmosphere of dry nitrogen with H_2O and O_2 at less than 0.1 ppm. All glassware was dried by flame-drying with subsequent cooling under 10^{-3} mbar vacuum followed by repeated alternate evacuation and purging with nitrogen. Where necessary, THF, pentane, toluene, and DME were dried by passage through activated alumina, degassed prior to use, and stored over activated 3Å molecular sieves (THF and DME) or a potassium mirror (pentane and toluene). Deuterated solvent was distilled from a potassium mirror and degassed by three freeze-pump-thaw cycles and stored under nitrogen. FTIR spectra were recorded on a Bruker Tensor 27 spectrometer. $LnI_3THF_{3.5}$ (Ln = Y, Gd and Dy),[1] MCH_2Ph (M = Na, K, Rb),[2] SCS-H_2,[3] were prepared according to the literature. Dibenzo-18-crown-6 ether was obtained from commercial sources, dissolved in THF, dried over 3Å mol sieves for 48 hours, precipitated and thoroughly dried in vacuo before use. 2,2,2-crypt was obtained from commercial sources and used as received.

Preparation of [Dy(SCS)(SCSH)(THF)] (1Dy)

Toluene (40 ml) was added to a pre-cooled (–78 °C) mixture of $DyI_3(THF)_{3.5}$ (3.00 g, 3.77 mmol), KCH_2Ph (1.47 g, 11.31 mmol) and $SCSH_2$ (3.04 g, 6.79 mmol). The resulting orange suspension was slowly warmed to room temperature with stirring over 16 hours to afford a yellow solution. Toluene was removed *in vacuo* down to 20 ml and THF (20 ml) was added. After filtering away from residual KI, volatiles were removed *in vacuo* to produce a white powder. Washing with a combination of toluene (8 ml) and pentane (20 ml) produced a fine colourless powder, which was recrystallised from hot toluene to afford colourless crystals of **1Dy**. Yield: 3.8 g, 90% with respect to dysprosium. Anal. Calcd for $C_{54}H_{49}DyOP_4S_4$: C, 57.46;

H, 4.38; N, 0%. Found: C, 57.93; H, 4.76; N, 0%. FTIR v/cm^{-1} (ATR): 3020 (w), 2633 (w), 2320 (w), 2323 (w), 2284 (w), 2161 (w), 2047 (w), 2037 (w), 2008 (vw), 1980 (w), 1435 (m), 1297 (s), 1156 (w), 1099 (m), 1013 (w), 921 (m), 860 (w), 780 (w), 738 (m), 697 (s), 587 (vs), 528 (w), 493 (s), 444 (w), 400 (m).

Preparation of [Y(SCS)(SCSH)(THF)] (1Y)

Prepared as per the method of **1Dy**. A mixture of $YI_3(THF)_{3.5}$ (3.00 g, 3.92 mmol), KCH_2Ph (1.53 g, 11.77 mmol), and $SCSH_2$ (3.16 g, 7.06 mmol) gave colourless crystals after work-up and cooling to room temperature from hot toluene. Yield: 3.7 g, 90% with respect to yttrium. Anal. Calcd for $C_{54}H_{49}OP_4S_4Y$: C, 61.48; H, 4.68; N, 0%. Found: C, 61.12; H, 4.76; N, 0%. FTIR v/cm^{-1} (ATR): 3047 (w), 2634 (w), 2360 (w), 2323 (w), 2284 (w), 2150 (w), 2050 (w), 2040 (w), 2012 (vw), 1980 (w), 1435 (m), 1297 (s), 1154 (w), 1098 (m), 1015 (w), 918 (m), 860 (w), 780 (w), 680 (s), 592 (vs), 528 (w), 493 (s), 444 (w). ^{1}H NMR (C_6D_6, 298 K): δ 1.13 (4H, m, THF) 2.96 (1H, d, J_{CH} = 1.6 Hz, C(*H*)P_2), 3.73 (4H, m, THF), 6.81 (12H, m, *para*/*meta*-Ar-H), 7.04 (12H, m, *para*/*meta*-Ar-H), 7.75 (8H, m, *ortho*-Ar-H), 7.89 (8H, m, *ortho*-Ar-H). ^{13}C{^{1}H} NMR (C_6D_6, 298K): δ 26.17 (THF), 43.89 (t, $^2J_{PC}$ = 5.8 Hz, Y*C*(H)P_2), 61.47 (THF), 126.13 (Ar-C), 127.66 (Ar-C), 127.71 (Ar-C), 127.76 (Ar-C), 128.35 (Ar-C), 129.17 (Ar-C), 130.87 (t, $^2J_{PC}$ = 5.8 Hz, Y*C*P_2), 131.22 (*ipso*-Ar-C), 131.26 (*ipso*-Ar-C). ^{31}P{^{1}H} NMR (C_6D_6, 298 K): δ 13.40 (d, $^2J_{YP}$ = 13.13 Hz, YC*P*$_2$), 33.27 (d, $^2J_{YP}$ = 6.75 Hz, YC(H)*P*$_2$).

Preparation of [Gd(SCS)(SCSH)(THF)] (1Gd)

Prepared as [Dy(SCS)(SCSH)(THF)]. $GdI_3(THF)_{3.5}$ (0.41 g, 0.50 mmol), $K(CH_2Ph)$ (0.20 g, 1.50 mmol) and SCS-H_2 (0.40 g, 0.90 mmol) gave colourless crystals after cooling from hot toluene. Yield: 0.28 g, 60% with respect to $GdI_3(THF)_{3.5}$. Anal. Calcd for $C_{54}H_{49}GdOP_4S_4$: C, 57.73; H, 4.40; N, 0%. Found: C, 57.80; H, 4.29; N, 0%. FTIR v/cm^{-1} (ATR): 3042 (vw), 2650

(w), 2351 (w), 2313 (vw), 2260 (w), 2149 (w), 2083 (w), 1999 (vw), 1971 (w), 1442 (m), 1300 (s), 1145 (w), 1020 (m), 999 (w), 929 (w), 860 (w), 758 (w), 685 (s), 590 (vs), 534 (w), 501 (s), 445 (w).

Preparation of [Dy(SCS)$_2$][Dy(SCS)$_2$K$_2$(DME)$_4$] (2Dy)

DME (15 ml) was added to a pre-cooled (–78 °C) mixture of **1Dy** (0.525 g, 0.50 mmol) and KCH$_2$Ph (0.065 g, 0.50 mmol). The resulting yellow suspension was allowed to warm up slowly over 16 hours to afford a yellow solution with a cream-coloured precipitate. The precipitate was warmed into solution and immediately filtered, which produced colourless crystals of **2Dy** upon cooling the mother liquor to room temperature. Yield: 0.52 g, 81%. Anal. Calcd for $C_{116}H_{120}Dy_2K_2O_8P_8S_8$: C, 54.64; H, 4.74; N, 0%. Found: C, 54.17; H, 4.90; N, 0%. FTIR v/cm^{-1} (ATR): 3045 (w), 2898 (w), 2818 (w), 1978 (w), 1476 (w), 1431 (w), 1319 (m), 1319 (m), 1254 (s), 1097 (s), 1078 (s), 1023 (m), 850 (w), 742 (m), 707 (m), 691 (s), 660 (m), 617 (w), 573 (s), 560 (s), 499 (s), 485 (s), 460 (s), 422 (w).

Preparation of [Y(SCS)$_2$][Y(SCS)$_2$K$_2$(DME)$_4$] (2Y)

Prepared as per the method of **2Dy**. A mixture of **1Y** (0.450 g, 0.46 mmol) and KCH$_2$Ph (0.060 g, 0.46 mmol) gave **2Y** as colourless crystals after work-up and cooling to room temperature from a hot filtration in DME. Yield: 0.49 g, 88%. Anal. Calcd for $C_{116}H_{120}K_2O_8P_8S_8Y_2$: C, 57.99; H, 5.03; N, 0%. Found: C, 58.02; H, 5.06; N, 0%. ^{1}H NMR (THF/C_6D_6, 298K): 3.12 (24H, s, OCH$_3$), 3.32 (16H, s, OCH$_2$), 6.96 (48H, m, *para/meta*-Ar-H), 7.86 (32H, m, *ortho*-Ar-H). ^{13}C{^{1}H} NMR (THF/C_6D_6, 298K): δ 63.43 (OCH$_3$), 70.02 (OCH$_2$), 126.00 (Ar-C), 128.07 (Ar-C), 129.89 (Ar-C), 130.63 (t, J_{PC} = 5.8 Hz, Y*C*P$_2$), 147.51 (s, *ipso*-Ar-C) ppm. ^{31}P{^{1}H} NMR (THF/C_6D_6, 298 K): δ 13.33 (d, $^2J_{YP}$ = 10.6 Hz, YC*P*$_2$). FTIR v/cm^{-1} (ATR): 3049 (w), 2889 (w), 2816 (w), 1584 (w), 1476 (w), 1433 (w), 1316 (m), 1299 (m), 1250 (s),

1087 (s), 1067 (s), 1020 (m), 910 (w), 850 (w), 780 (w),, 736 (m), 707 (m), 682 (s), 656 (m), 616 (w), 562 (s), 560 (s), 500 (s), 480 (s), 422 (w), 414 (s).

Preparation of [Dy(SCS)$_2$][Na(DME)$_3$] (3Dy)

DME (15 ml) was added to a pre-cooled (–78 °C) mixture of **1Dy** (0.525 g, 0.50 mmol) and $NaCH_2Ph$ (0.065 g, 0.50 mmol). The resulting orange suspension was allowed to warm up slowly over 16 hours to afford a yellow solution with cream-coloured precipitate. The precipitate was warmed into solution and immediately filtered, which produced colourless crystals of **2Dy** upon cooling the mother liquor to room temperature. Yield: 0.49 g, 73%. Anal. Calcd for $C_{62}H_{70}DyNaO_6P_4S_4$: C, 55.21; H, 5.23; N, 0%. Found: C, 54.96; H, 5.29; N, 0%. FTIR v/cm^{-1} (ATR): 3018 (s), 2643 (w), 2323 (w), 2286 (w), 2161 (m), 2072 (m), 2047 (s), 2035 (s), 2008 (m), 1978 (vs), 1821 (w), 1431 (w), 1305 (w), 1262 (m), 1242 (m), 1082 (m), 856 (w), 689 (s), 575 (s), 485 (s), 454 (w), 401 (s).

Preparation of [Y(SCS)$_2$][Na(DME)$_3$] (3Y)

Prepared as per the method of **3Dy**. A mixture of **1Y** (0.500 g, 0.47 mmol) and $NaCH_2Ph$ (0.053 g, 0.47 mmol) gave **3Y** as colourless crystals after work-up and cooling to room temperature from a hot filtration in DME. Yield: 0.54 g, 91%. Anal. Calcd for $C_{62}H_{70}NaO_6P_4S_4Y$: C, 58.39; H, 5.53; N, 0%. Found: C, 58.72; H, 5.66; N, 0%. ^{1}H NMR (THF/C_6D_6, 298K): 3.12 (18H, s, OCH_3), 3.31 (12H, s, OCH_2), 6.94 (24H, m, *meta/para*-Ar-H), 7.74 (16H, m, *ortho*-Ar-H). $^{13}C\{^1H\}$ NMR (C_6D_6, 298K): δ 65.55 (DME), 69.76 (DME), 126.47 (Ar-C), 128.05 (Ar-C), 128.82 (Ar-C), 131.29 (t, J_{PC} = 5.8 Hz, Y*C*P_2), 143.48 (d, *ipso*-Ar-C) ppm. $^{31}P\{^1H\}$ NMR (THF/C_6D_6, 298 K): δ 13.32 (d, $^2J_{YP}$ = 10.6 Hz, YC*P*$_2$). FTIR v/cm^{-1} (ATR): 3047 (w), 2920 (w), 1476 (w), 1433 (m), 1252 (s), 1080 (s), 1025 (w), 856 (m), 801 (w), 736 (w), 705 (m), 689 (s), 664 (m), 620 (w), 569 (s), 513 (m), 483 (s), 416 (w).

Preparation of [Gd(SCS)$_2$][Na(DME)$_3$] (3Gd)

DME (15 ml) was added to a pre-cooled (-78°C) mixture of Gd(SCS)(SCSH)(THF) (0.63g, 0.6 mmol) and Na(CH_2Ph) (0.068 g, 0.6 mmol). The resulting yellow suspension was allowed to warm up slowly over 16 hours to afford a pale-yellow solution with cream precipitate. Colourless crystals of [Gd(SCS)$_2$][Na(DME)$_3$] were obtained from a concentrated solution at -30°C. Yield: 20%. Anal. Calcd for $C_{62}H_{70}GdNaO_6P_4S_4$: C, 55.42; H, 5.25; N, 0%. Found: C, 54.97; H, 4.81; N, 0%. FTIR v/cm^{-1} (ATR): 3043 (w), 2962 (w), 2821 (w), 1475 (w), 1433 (m), 1366 (w), 1259 (s), 1171 (w), 1083 (s), 1025 (s), 973 (w), 858 (m), 799 (s), 736 (m), 688 (s), 665 (m) 566 (s), 513 (m), 485 (s) 455 (m).

Preparation of [Dy(SCS)$_2$][K(2,2,2-crypt)] (4Dy)

THF (15ml) was added to a pre-cooled (–78 °C) mixture of **1Dy** (0.525 g, 0.50 mmol) and KCH_2Ph (0.065 g, 0.50 mmol) and 2,2,2-cryptand (0.188 g, 0.50 mmol). The resulting yellow suspension was allowed to warm up slowly over 16 hours to afford a yellow solution. Solvents were removed *in vacuo* and THF was added (5 ml). Dropwise addition of pentane (5 ml) with mixing then gave **4Dy** as colourless crystals after standing for 5 minutes. Yield: 0.55 g, 72%. Anal. Calcd for $C_{72}H_{84}DyKN_2O_7P_4S_4$: C, 56.04; H, 5.49; N, 1.82%. Found: C, 56.33; H, 5.33; N, 2.00%. FTIR v/cm^{-1} (ATR): 3047 (w), 3016 (w), 2867 (w), 2068 (w), 2033 (w), 2004 (w), 1976 (w), 1474 (w), 1431 (m), 1321 (s), 1295 (s), 1260 (m), 1172 (w), 1129 (m), 1095 (s), 1072 (s), 1025 (m), 950 (m), 932 (m), 828 (w), 742 (m), 691 (s), 652 (m), 566 (s), 495 (s), 452 (s), 409 (s).

Preparation of [Y(SCS)$_2$(THF)][K(2,2,2-crypt)] (4Y)

Prepared as per the method of **4Dy**. A mixture of **1Y** (0.536 g, 0.50 mmol), KCH_2Ph (0.065 g, 0.50 mmol), and 2,2,2-crypt (0.188g, 0.50 mmol) gave **4Y** as colourless crystals after work-up

and storing in an equal parts solution of pentane:THF (10 ml) at −30 °C for 3 days. Yield: 0.52 g, 81%. Anal. Calcd for $C_{62}H_{70}NaO_6P_4S_4Y$: C, 58.39; H, 5.53; N, 0%. Found: C, 58.72; H, 5.66; N, 0%. ^{1}H NMR (THF/C_6D_6, 298K): δ 2.11 (12H, t, $^2J_{CH}$ = 6.3, 3.1 Hz, NC*H*$_2$CH$_2$O), 3.09 (12H, t, $^2J_{CH}$ = 4.5 Hz, NCH$_2$C*H*$_2$O), 3.16 (12H, s, OCH$_2$), 7.01 (24H, m, para/*meta*-Ar-H), 8.06 (16H, m, *ortho*-Ar-H). ^{13}C{^{1}H} NMR (C_6D_6, 298K): δ 22.1 (THF), 55.5 (N*C*H$_2$CH$_2$O), 65.5 (NCH$_2$*C*H$_2$O), 71.5 (THF), 74.1 (O*C*H$_2$*C*H$_2$O), 122.7 (Ar-C), 128.9 (Ar-C), 132.1 (t, J_{PC} = 5.9 Hz, Y*C*P$_2$), 135.3 (Ar-C), 150.5 (d, *ipso*-Ar-C) ppm. ^{31}P NMR (C_6D_6/THF), 298 K): δ 13.32 (d, $^2J_{YP}$ = 10.6 Hz, YCP$_2$). FTIR v/cm^{-1} (ATR): 3049 (w), 2868 (w), 2068 (w), 2035 (w), 2006 (w), 1976 (w), 1819 (w), 1472 (w), 1425 (m), 1321 (s), 1290 (s), 1260 (m), 1170 (w), 1120 (m), 1095 (s), 1071 (s), 1025 (m), 950 (m), 930 (m), 830 (w), 738 (m), 711(m), 703 (m), 691 (s), 652 (m), 561 (s), 495 (s), 442 (s), 409 (s).

[Dy(NCN)$_2$][K(DB18C6)(THF)(Toluene)] (5Dy)

THF (15 ml) was added to a precooled (–78 °C) mixture of [Dy(NCN)(NCNH)] (2.55 g, 2.00 mmol) and K(CH$_2$Ph) (0.26 g, 2.00 mmol). The resulting orange suspension was allowed to slowly warm to room temperature and stirred for 3 hours to afford a yellow solution. Dibenzo-18-crown-6 (0.72 g, 2.00 mmol) in THF was then added and the resulting yellow solution stirred for 1 hour. The volatiles were removed *in vacuo* and the resulting yellow solid recrystallised from a hot toluene:THF mixture (5:0.5 ml) to afford yellow crystals of **5Dy** on storing at room temperature. Yield: 75%. Anal. Calcd for $C_{93}H_{116}DyKN_4O_7P_4Si_4$: C, 60.71; H, 6.36; N, 3.05%. Found: C, 60.27; H, 6.48; N, 3.12%. FTIR v/cm^{-1} (ATR): 3053 (w), 2941 (w), 2884 (w), 1594 (w), 1501 (m), 1453 (w), 1433 (w), 1382 (m), 1282 (w), 1241 (s), 1207 (m), 1124 (m), 1102 (s), 1061 (s), 955 (w), 940 (m), 827 (s), 736 (s), 715 (m), 695 (s), 660 (m), 633 (m), 594 (s), 521 (s), 492 (s), 413 (m).

Magnetic Measurements

Magnetic measurements were performed in the temperature range 1.8 to 300 K with a Quantum Design MPMS-XL7 SQUID magnetometer equipped with a 7 T magnet. Polycrystalline samples were sealed in borosilicate NMR tubes and restrained with a small amount of eicosane to avoid alignment during measurements. Calibrated blanks were employed to account for the diamagnetic contribution from the tube and eicosane, while Pascal's constants were used for the diamagnetic contribution of the complex. Due to the observation of strange out-of-equilibrium behaviour in ZFC/FC measurements, an additional sequence incorporated a 10 minute wait at each temperature before the measurement was performed, which resulted in the slower ZFC curves presented. Magnetisation decay measurements in zero-field were performed by saturating the sample at 100 K in a 2 T field, before cooling down to the desired temperature. The external field was set to 0 T and measurements were then collected for between 400 – 4000 s, until equilibrium was reached. The decay curves were fitted using a stretched exponential defined below in equation 4, fixing the M_0 value to the initial data point after zero external field was achieved.

$$M(t) = M_1 + (M_0 - M_1)\exp\left[-(t/\tau)^b\right] \quad (4)$$

Electron Paramagnetic Resonance

EPR spectra were collected at Q-band (33.95491 GHz) microwave frequency using a Bruker EMX300 spectrometer. The **3Gd** sample was a polycrystalline powder in a flame sealed quartz EPR tube. Collection was performed at 5 K using liquid helium cooling.

Ab initio calculations

Molcas 8.0[4] was used for CASSCF-SO calculations using the RASSCF/RASSI/SINGLE_ANISO approach. For all calculations, Dy^{III} was described using VTZP basis from the Molcas ANO-RCC library, the first coordination sphere is described with VDZP quality and all other atoms use a VDZ quality basis sets.[5,6] The active space for Dy^{III} was nine electrons in seven 4f orbitals in a state-averaged CASSCF calculation performed separately for 21 sextets, 224 quartets and 261 doublets. 21 sextets, 128 quartets and 130 doublets were subsequently mixed by SO coupling. Cholesky decomposition was performed with a threshold of 10^{-8} to save disk space.

X-ray diffraction data

Crystals were examined using either a) an Agilent Supernova diffractometer, equipped with an Eos CCD area detector and a Microfocus source with Mo Kα radiation (λ = 0.71073 Å), b) an Agilent Supernova diffractometer, equipped with either an Atlas/AtlasS2 or TitanS2 CCD area detector and mirror-monochromated Cu Kα radiation (λ = 1.5418 Å), or c) a Rigaku FR-X diffractometer, equipped with a HyPix 6000HE photon counting pixel array detector with mirror-monochromated Mo Kα (λ = 0.71073 Å) or Cu Kα (λ = 1.5418 Å) radiation. Intensities were integrated from a sphere of data recorded on narrow (0.5 or 1.0°) frames by ω rotation. Cell parameters were refined from the observed positions of all strong reflections in each data set. Either multi-scan or Gaussian grid face-indexed absorption corrections with a beam profile correction were applied. The structures were solved by direct methods using SHELXT[7] or Superflip[8] and the datasets were refined by full-matrix least-squares on all unique F2 values, with anisotropic displacement parameters for all non-hydrogen atoms, and with constrained riding hydrogen geometries; $U_{iso}(H)$ was set at 1.2 (1.5 for methyl groups) times U_{eq} of the parent atom. The largest features in final difference syntheses were close to heavy atoms and

were of no chemical significance. CrysAlisPro[9] was used for control and integration, and SHELXL[10] and Olex2[11] were employed for structure refinement. ORTEP-3[12] and POV-Ray[13] were employed for molecular graphics.

Figures

IR Data

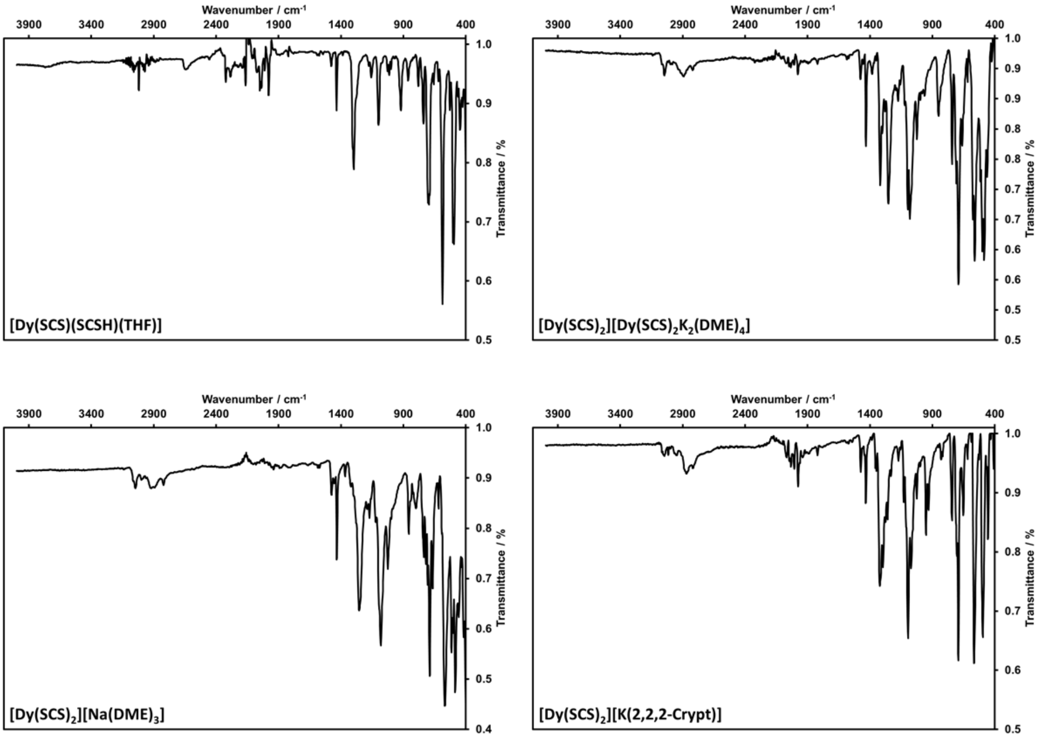

Figure S1. IR spectra of 1Dy-4Dy.

NMR Spectra

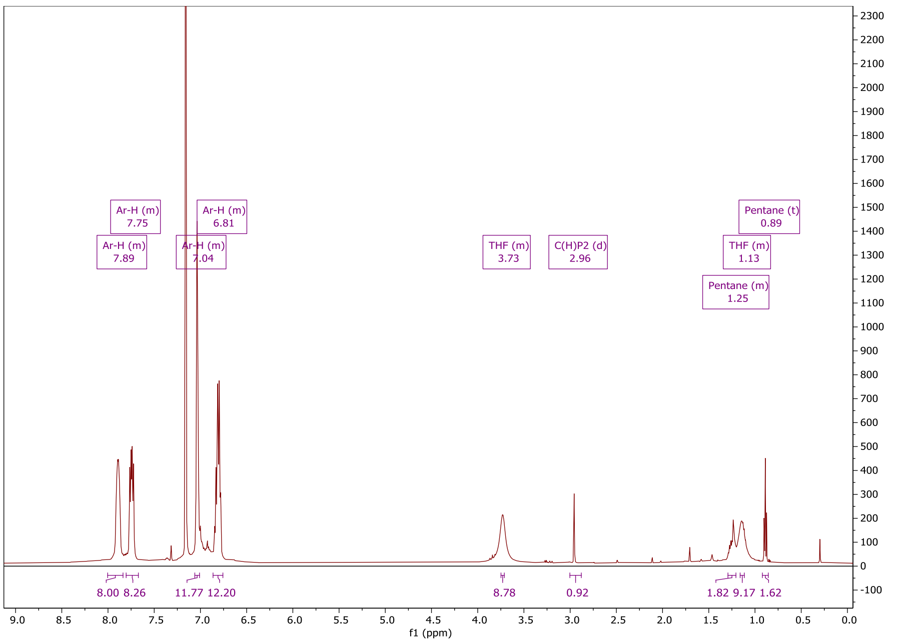

Figure S2. ***^{1}H NMR spectrum of 1Y.***

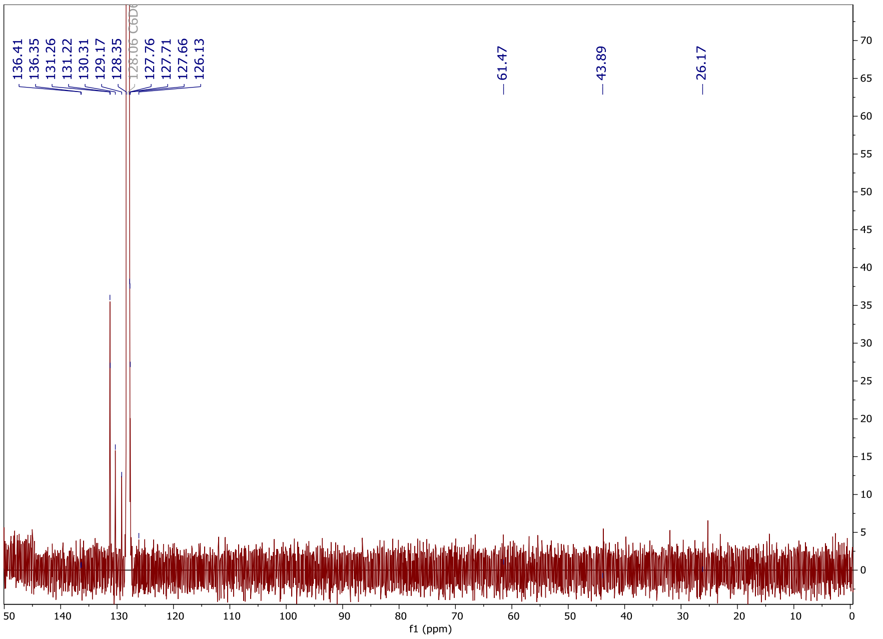

Figure S3. ***^{13}C NMR spectrum of 1Y.***

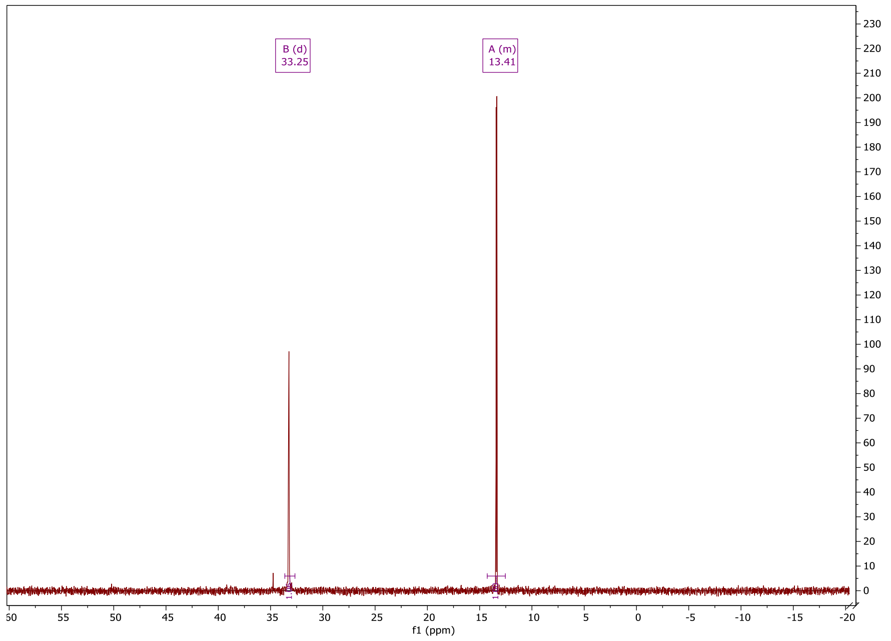

Figure S4. ^{31}P NMR spectrum of 1Y.

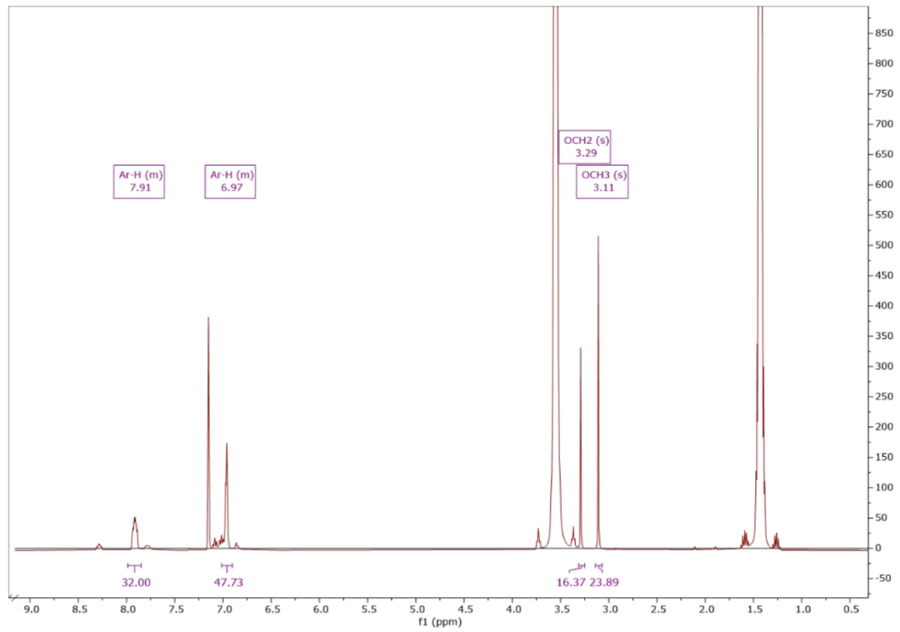

Figure S5. ^{1}H NMR spectrum of 2Y.

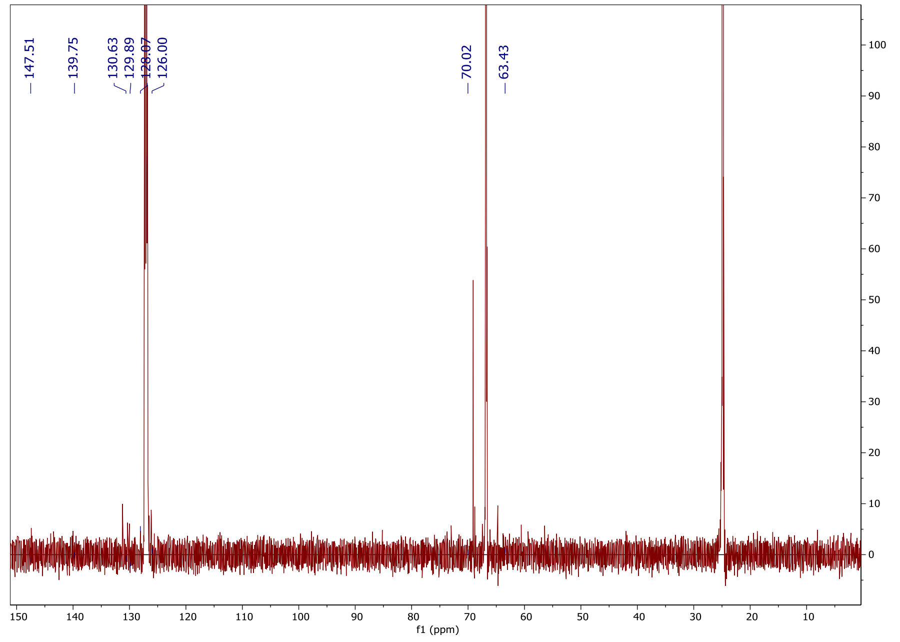

Figure S6. ***^{13}C NMR spectrum of 2Y.***

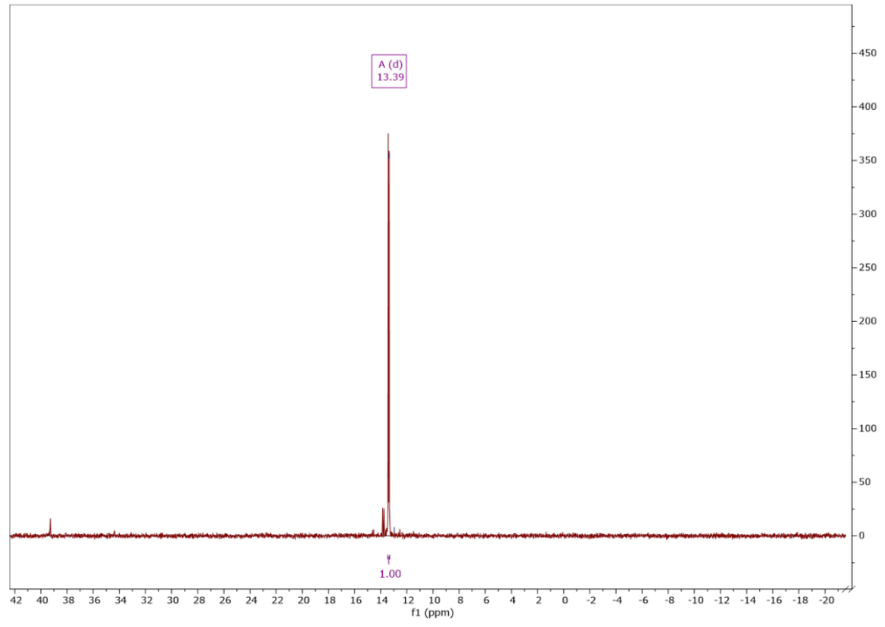

Figure S7. ***^{31}P NMR spectrum of 2Y.***

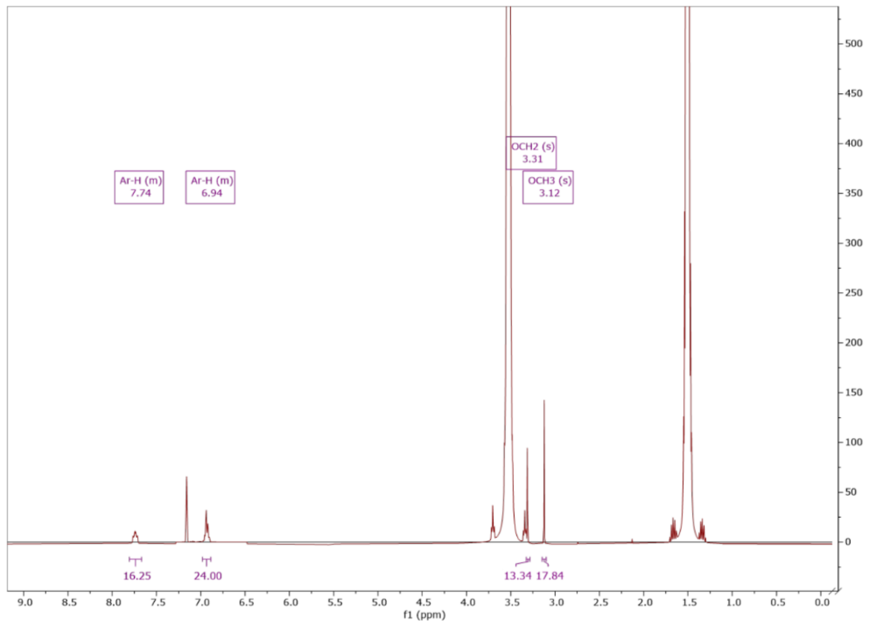

Figure S8. ^{1}H NMR spectrum of 3Y.

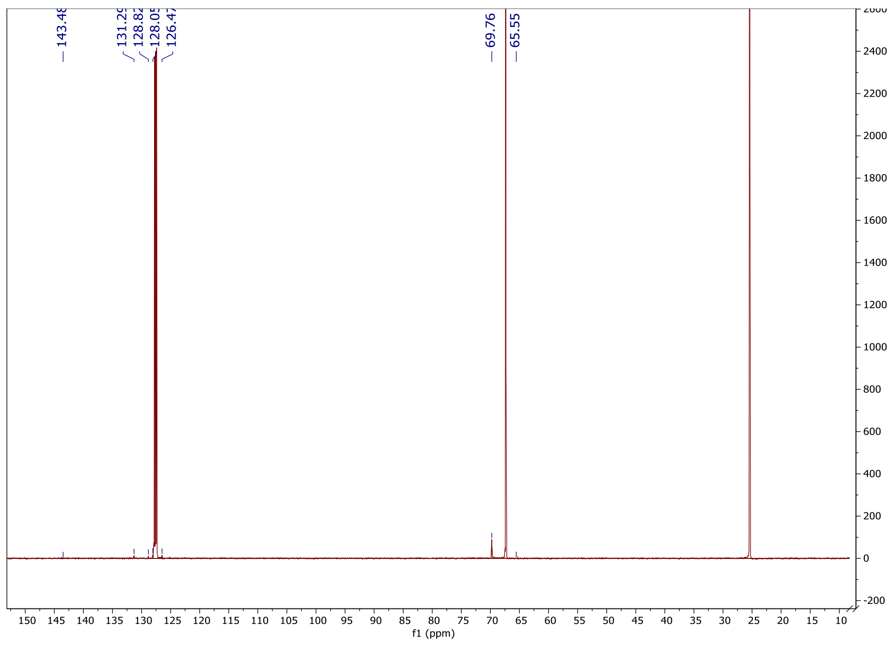

Figure S9. ^{13}C NMR spectrum of 3Y.

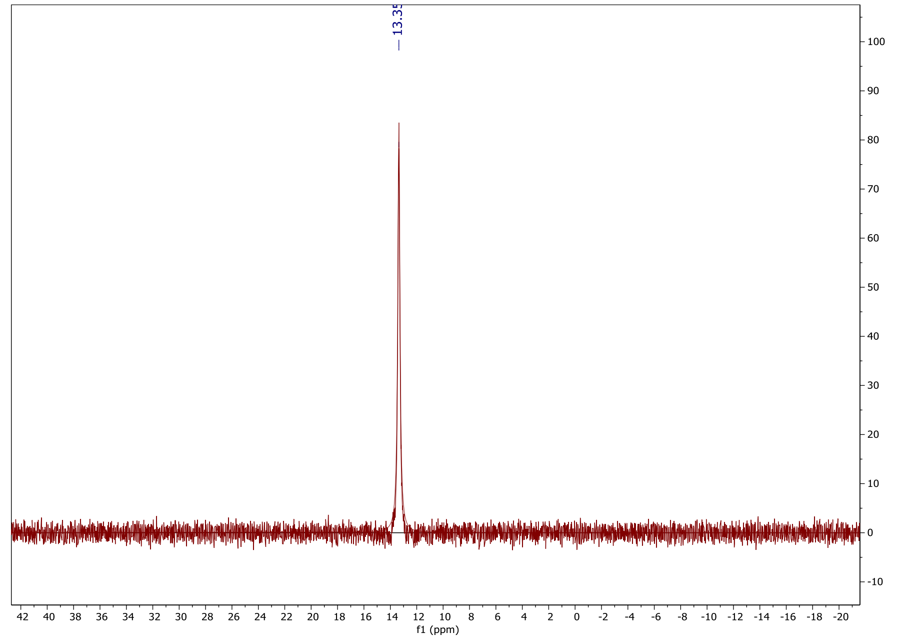

Figure S10. ^{31}P NMR spectrum of 3Y.

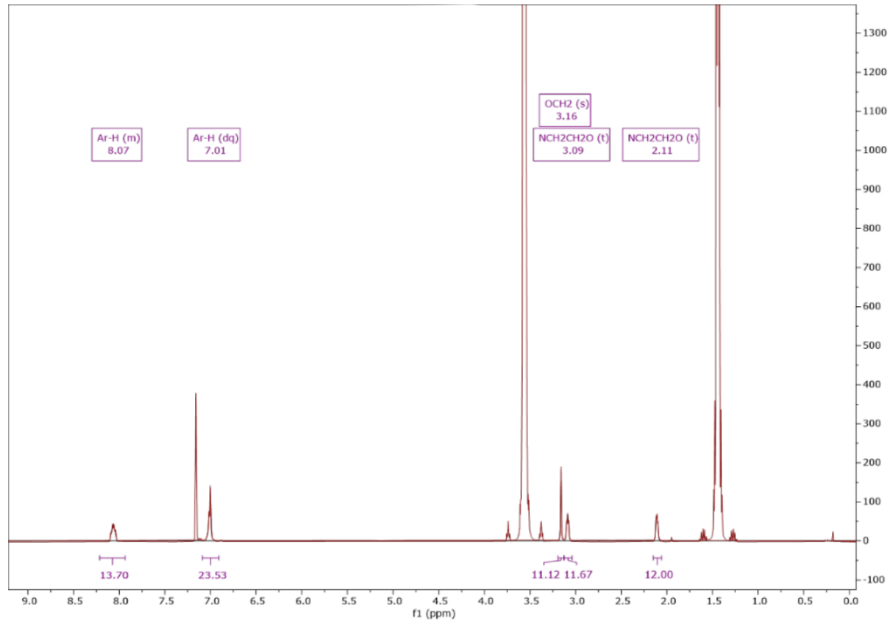

Figure S11. ^{1}H NMR spectrum of 4Y.

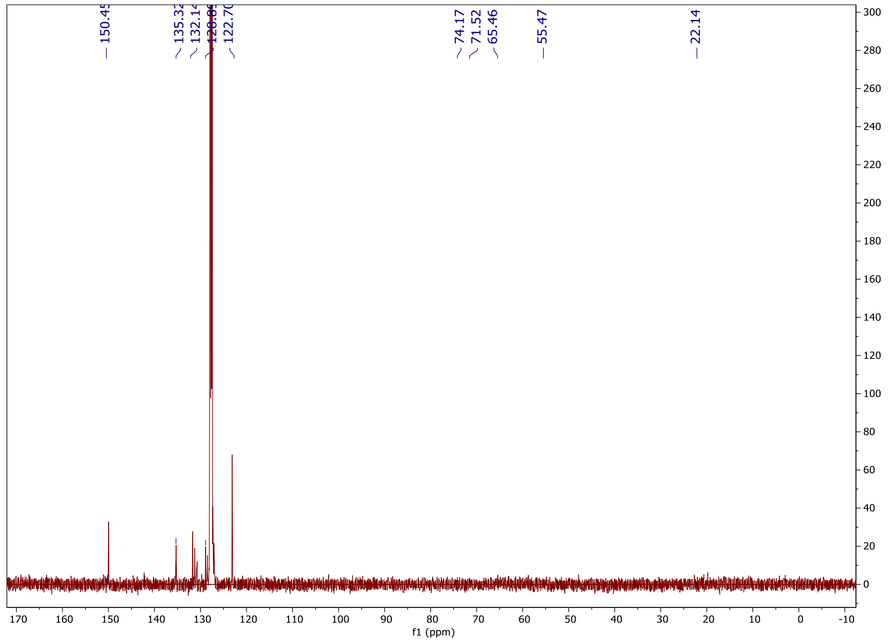

Figure S12. ^{13}C NMR spectrum of 4Y.

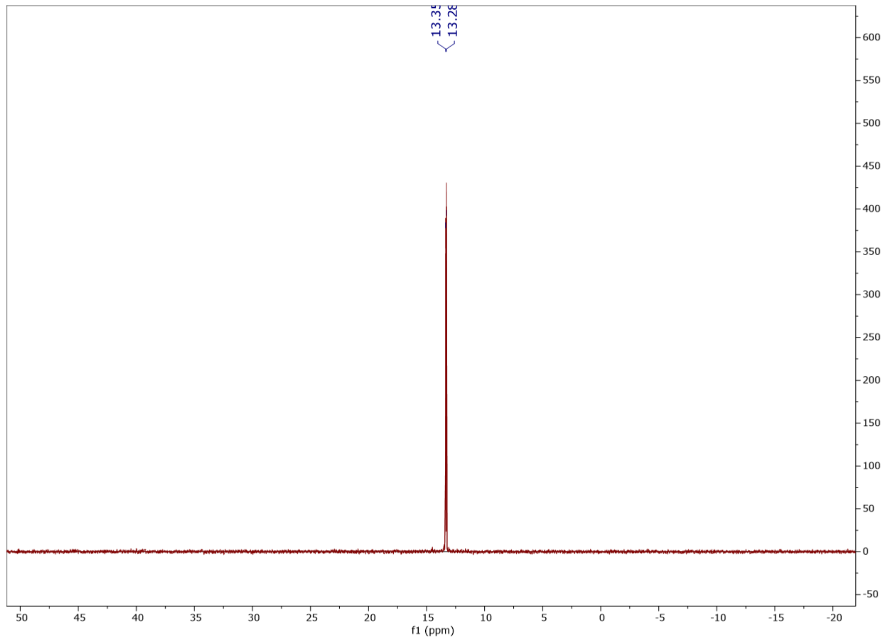

Figure S13. ^{31}P NMR spectrum of 4Y.

Crystallography

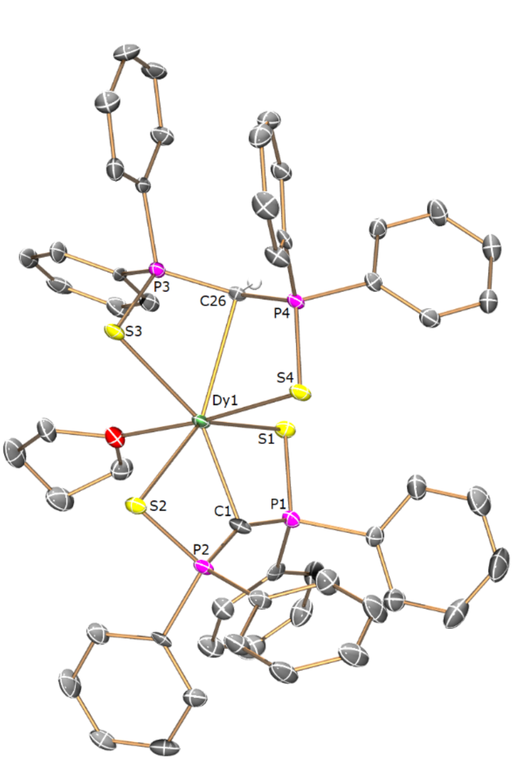

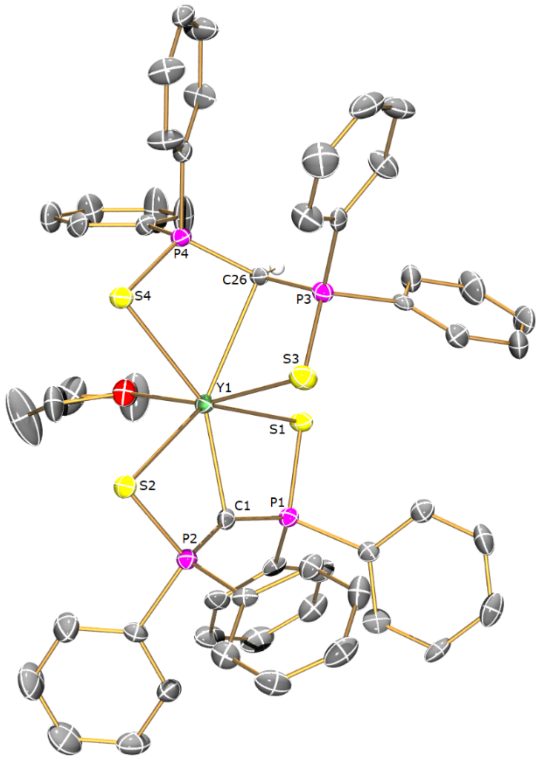

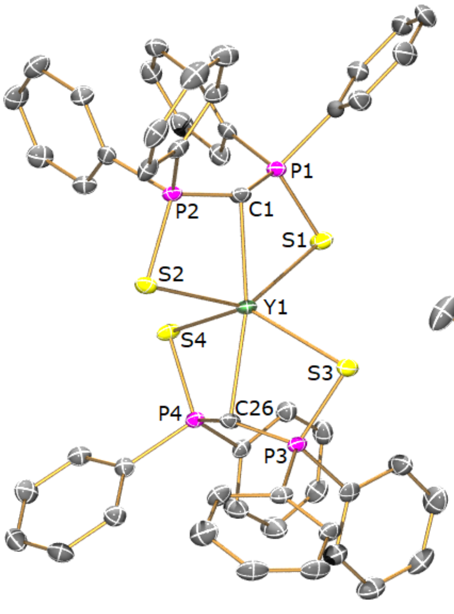

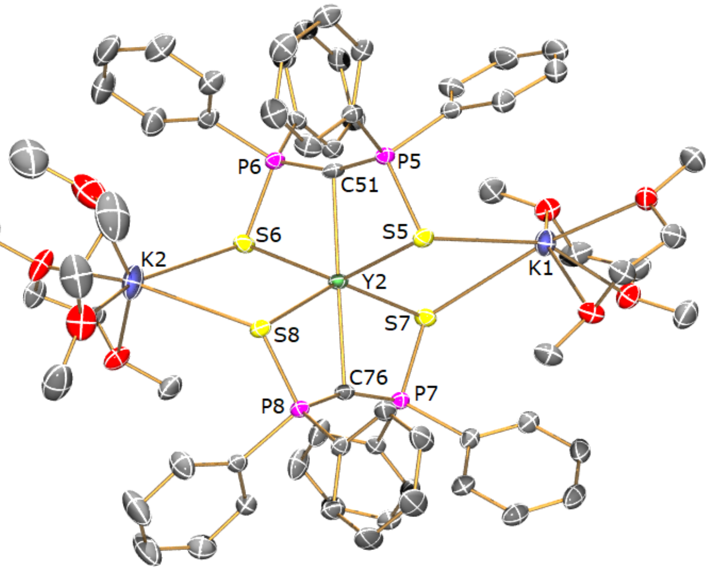

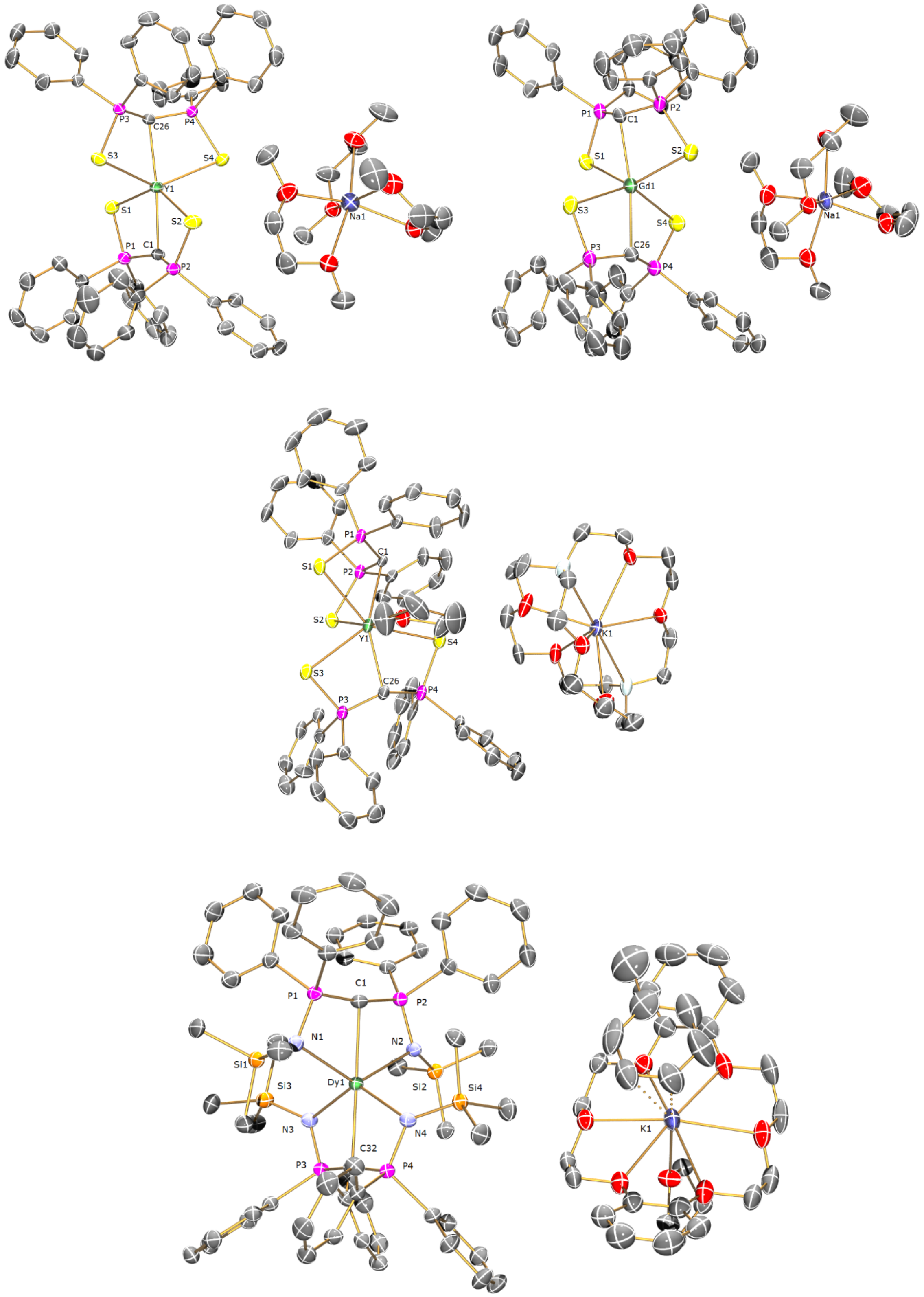

Figure S14. Left to right, top to bottom: solid state structures of 1Dy, 1Y, 2Y, 3Y, 3Gd, 4Y, and 5Dy. All at 40 % probability displacement ellipsoids with hydrogen atoms and disordered units excluded for clarity.

Magnetometry

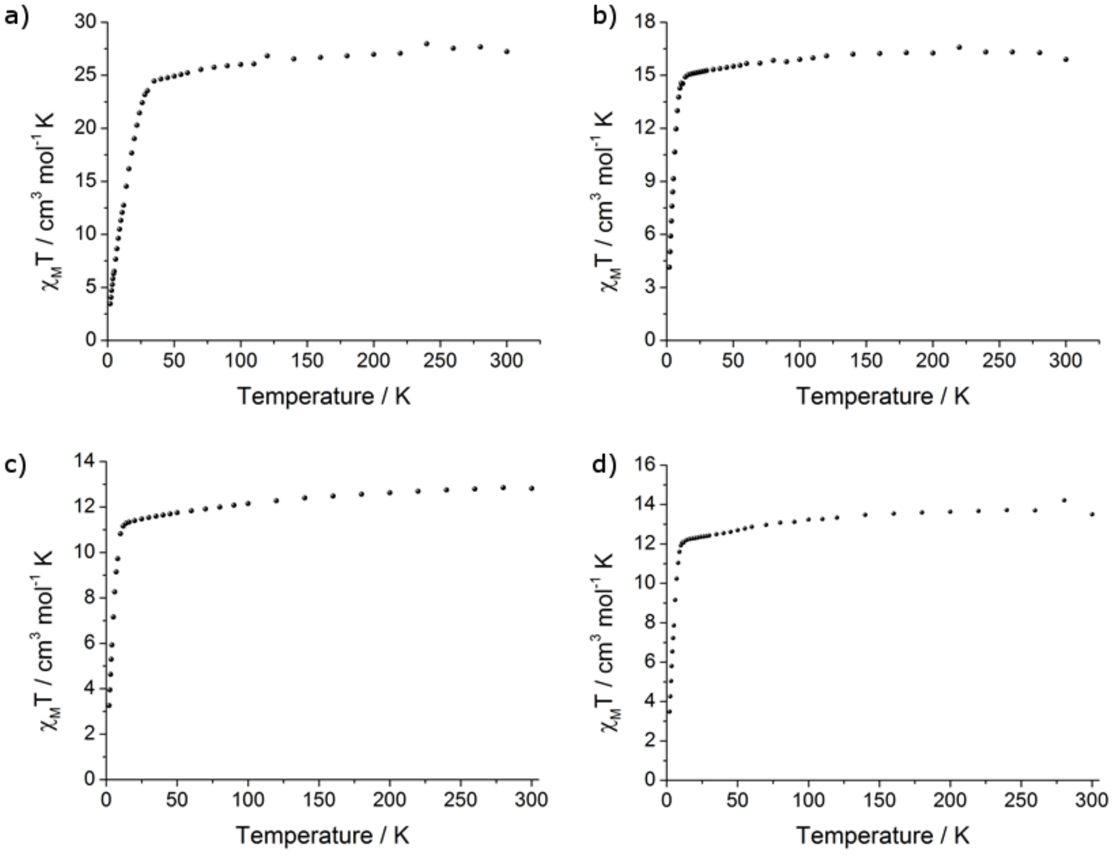

Figure S15. Magnetic susceptibility measurements of 2-5Dy (a-d respectively) performed with a 1000 Oe applied field in the temperature range 1.8 to 300 K.

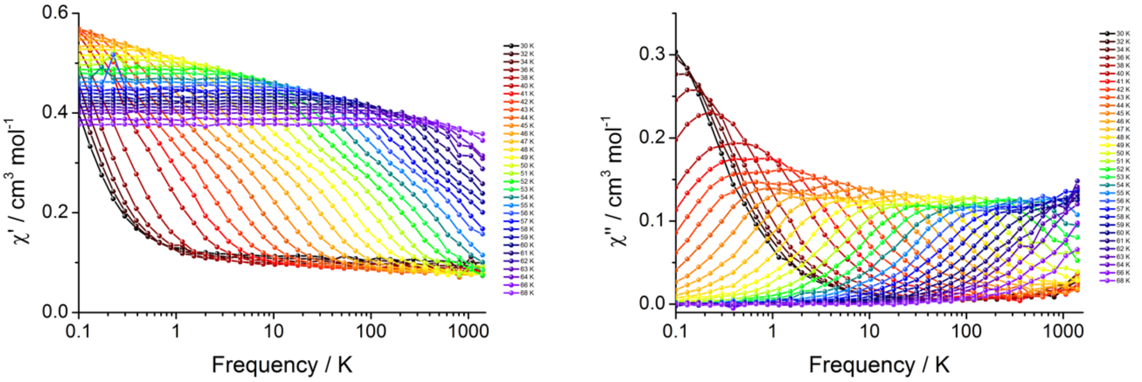

Figure S16. Alternating-current susceptibility data for 2Dy with the in-phase (left) and out-of-phase (right) signal depicted in the temperature range of 30 to 68 K.

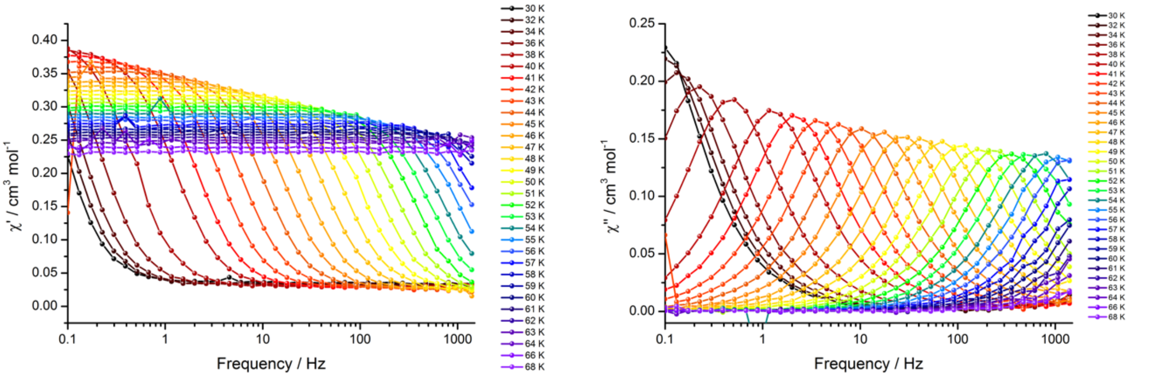

Figure S17. Alternating-current susceptibility data for 3Dy with the in-phase (left) and out-of-phase (right) signal depicted in the temperature range of 30 to 68 K.

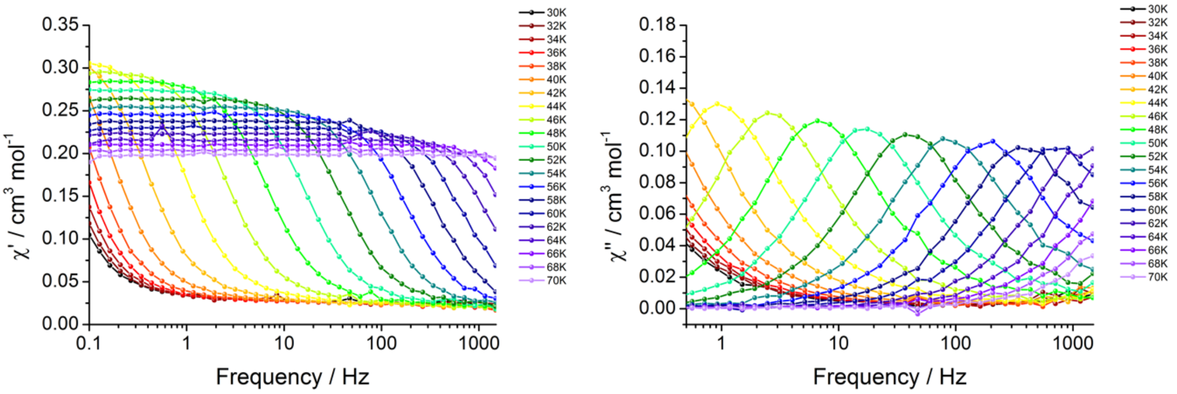

Figure S18. Alternating-current susceptibility data for 4Dy with the in-phase (left) and out-of-phase (right) signal depicted in the temperature range of 30 to 70 K.

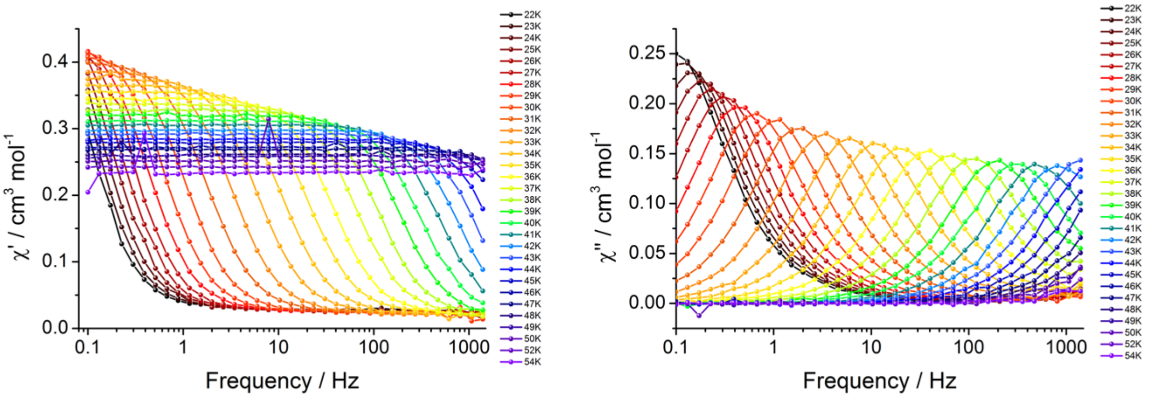

Figure S19. Alternating-current susceptibility data for 5Dy with the in-phase (left) and out-of-phase (right) signal depicted in the temperature range of 22 to 54 K.

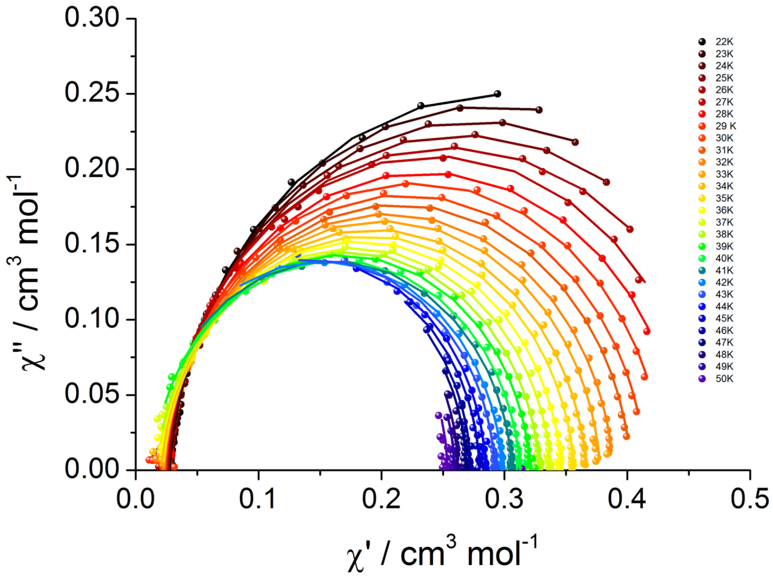

Figure S20. Cole-Cole data for 5Dy from 22 to 50 K showing experimental data (points) and fitted curves (lines).

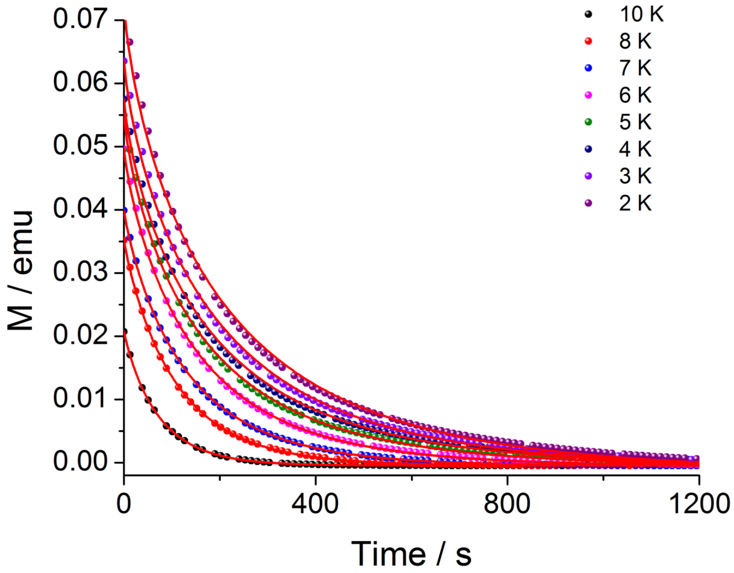

Figure S21. DC magnetisation decay measurements performed on 2Dy by saturating the sample in a 20 kOe applied magnetic field and then measuring the remanent magnetisation in a 0 applied field. Temperatures indicated in inset and red lines depict fits using exponential decay curve equation 3.

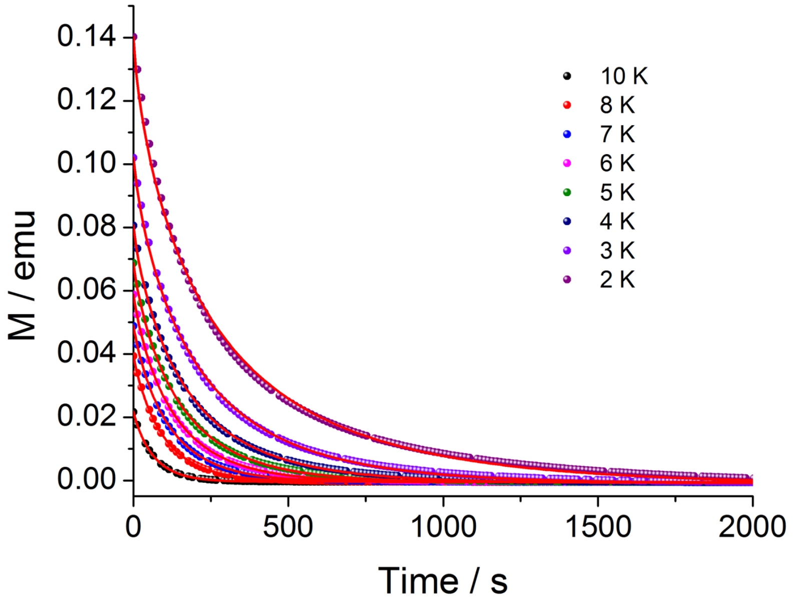

Figure S22. DC magnetisation decay measurements performed on 3Dy by saturating the sample in a 20 kOe applied magnetic field and then measuring the remanent magnetisation in a 0 applied field. Temperatures indicated in inset and red lines depict fits using exponential decay curve equation 3.

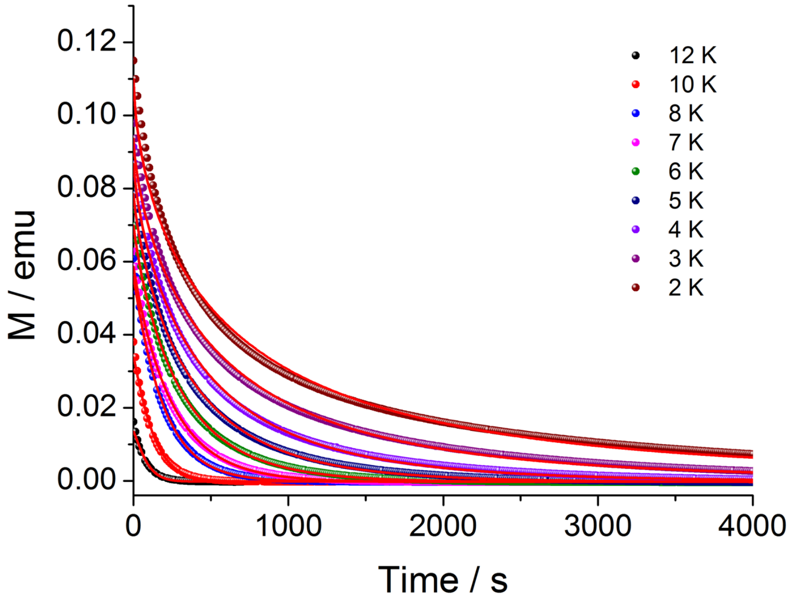

Figure S23. DC magnetisation decay measurements performed on 4Dy by saturating the sample in a 20 kOe applied magnetic field and then measuring the remanent magnetisation in a 0 applied field. Temperatures indicated in inset and red lines depict fits using exponential decay curve equation 3.

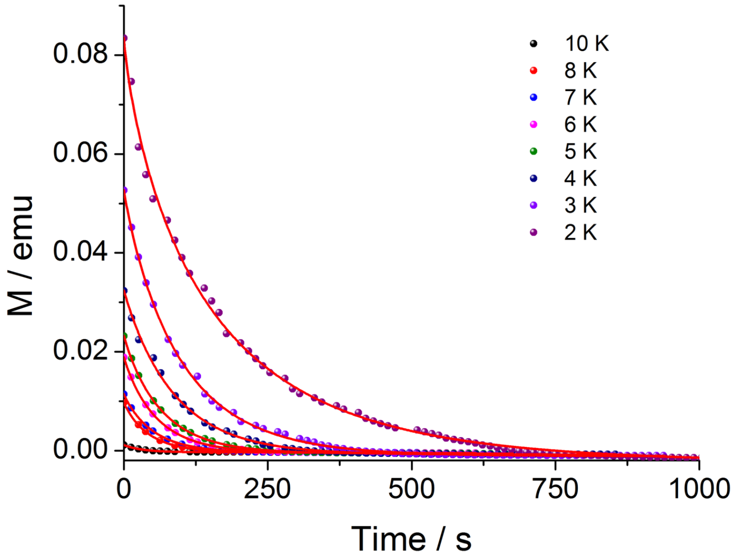

Figure S24. DC magnetisation decay measurements performed on 5Dy by saturating the sample in a 20 kOe applied magnetic field and then measuring the remanent magnetisation in a 0 applied field. Temperatures indicated in inset and red lines depict fits using exponential decay curve equation 3.

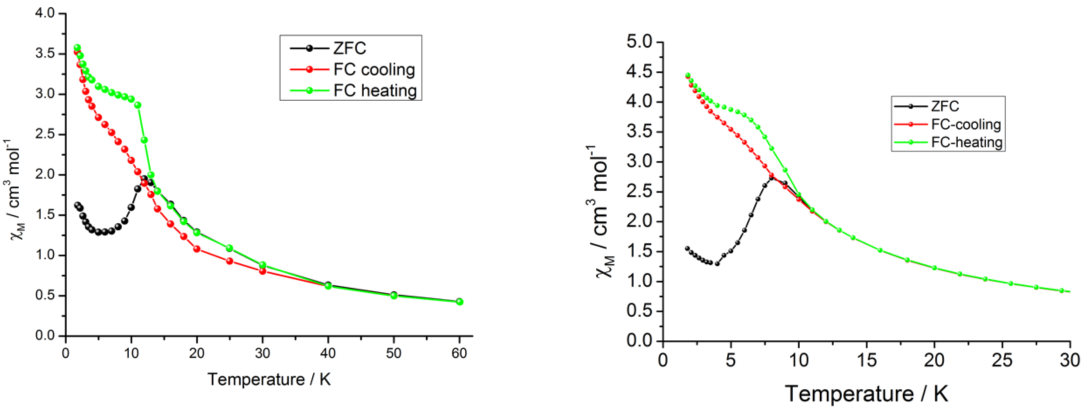

Figure S25. Zero-field cooled (black) and field cooled cooling (red) and heating (green) susceptibility data for 2Dy measured in a 1000 Oe applied field, showing bifurcation from 30 K and ZFC peak at 12 K (left) and bifurcation from 12 K and ZFC peak at 8 K (right). The high T_{IRREV} is likely due to temperature equilibration issues during the measurement. Average sweep rate of ~ 0.38 K / min (left) and ~ 0.031 K / min (right).

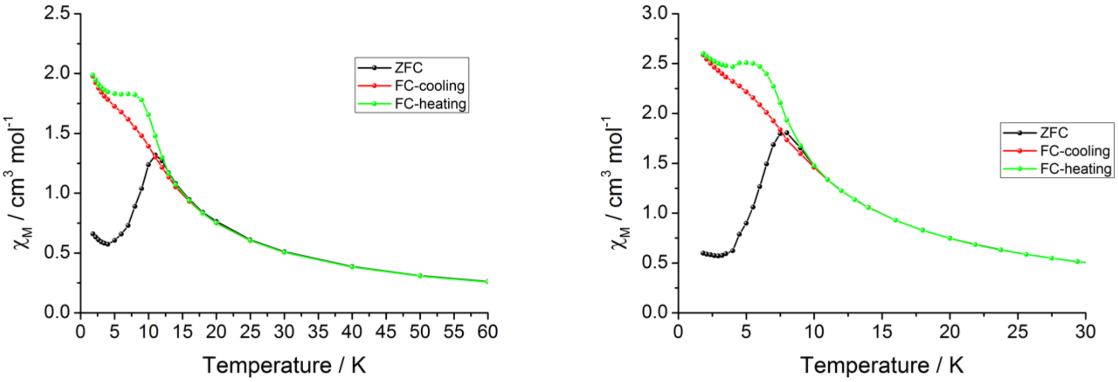

Figure S26. *Zero-field cooled (black) and field cooled cooling (red) and heating (green) susceptibility data for* ***3Dy*** *measured in a 1000 Oe applied field, showing bifurcation from 14 K and ZFC peak at 11 K (left) and bifurcation from 10 K and ZFC peak at 7.5 K (right). The high T_{IRREV} is likely due to temperature equilibration issues during the measurement. Average sweep rate of ~ 0.32 K / min (left) and ~0.031 K / min (right).*

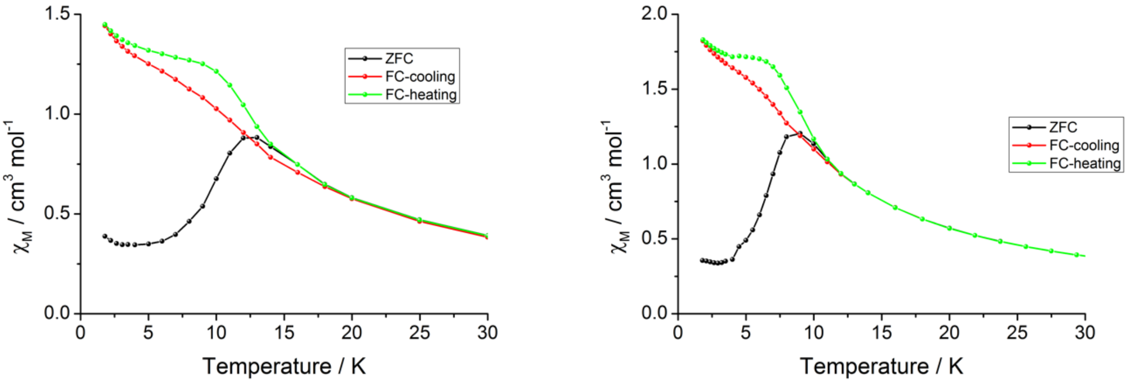

Figure S27. *Zero-field cooled (black) and field cooled cooling (red) and heating (green) susceptibility data for* ***4Dy*** *measured in a 1000 Oe applied field, showing bifurcation from 18 K and ZFC peak at 13 K (left) and bifurcation from 12 K and ZFC peak at 9 K (right). The high T_{IRREV} is likely due to temperature equilibration issues during the measurement. Average sweep rate of ~ 0.36 K / min (left) and ~ 0.031 K / min (right).*

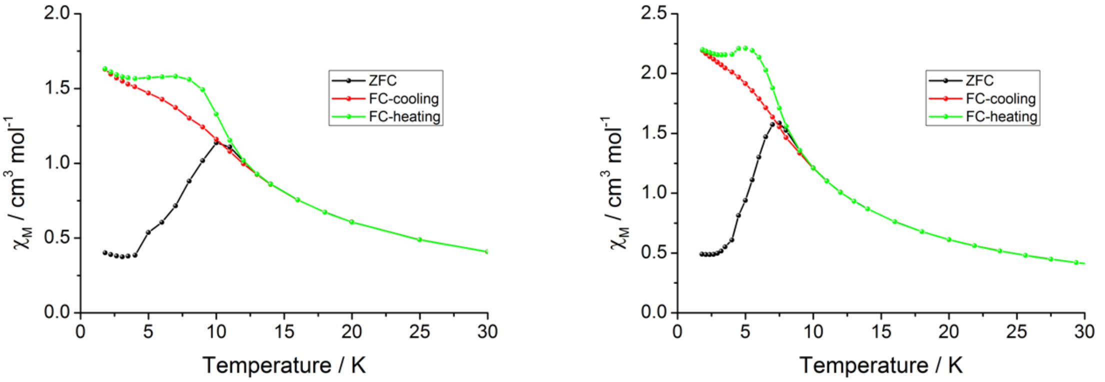

Figure S28. Zero-field cooled (black) and field cooled cooling (red) and heating (green) susceptibility data for 5Dy measured in a 1000 Oe applied field, showing bifurcation from 13 K and ZFC peak at 10 K (left) and bifurcation from 9 K and ZFC peak at 7.5 K (right). The high T_{IRREV} is likely due to temperature equilibration issues during the measurement. Average sweep rate of ~ 0.28 K / min (left) and ~ 0.031 K / min (right).

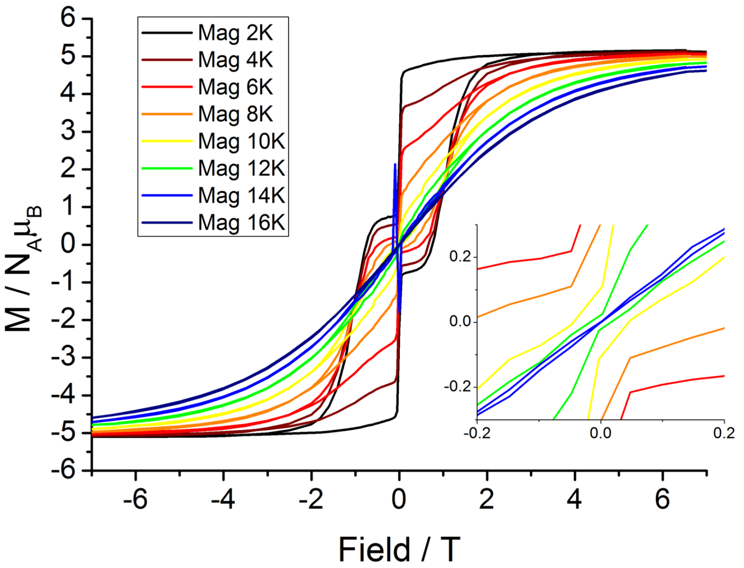

Figure S29. Magnetic hysteresis measurements performed on 5Dy from 1.8 to 16 K with a sweep rate of ~14 Oe/s. Inset shows a zoom around 0 field to show open hysteresis loops which close around 12 K.

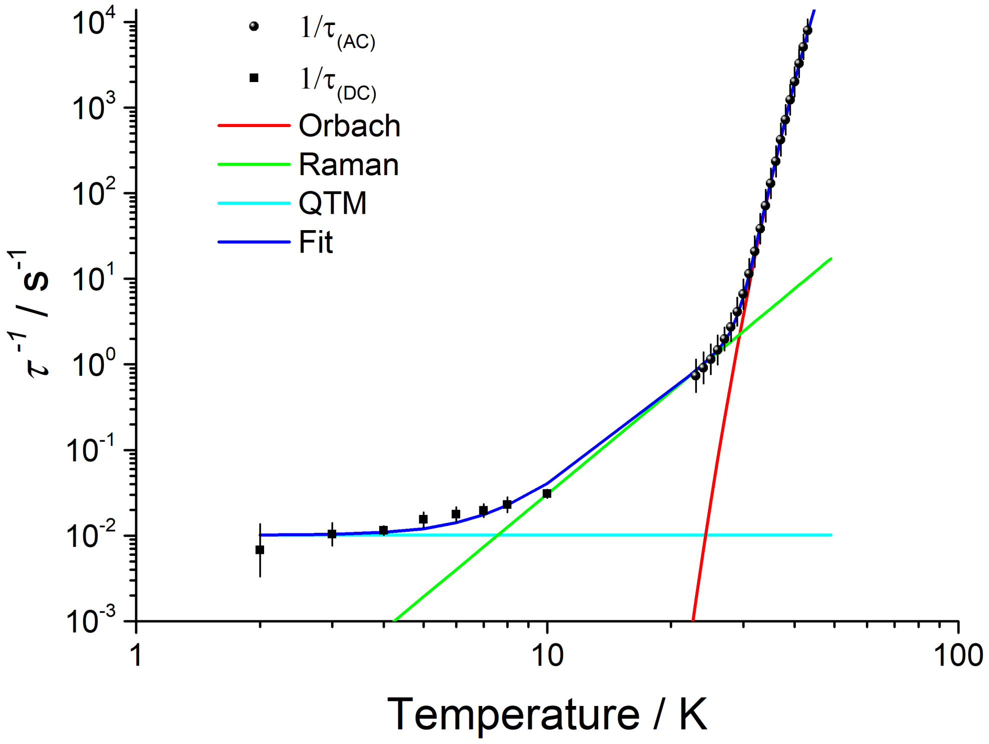

Figure S30. Fitted relaxation rate data for 5Dy sample using Orbach, Raman and QTM parameters reported in text. Orbach regime (red), Raman (green), QTM (cyan) and overall fit (blue) shown as solid lines.

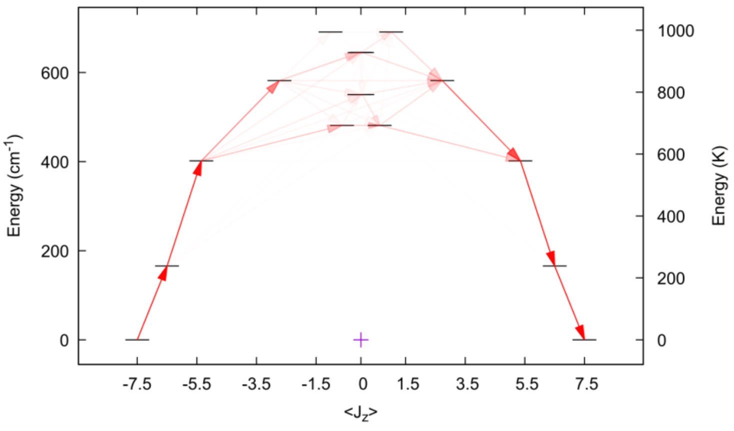

Figure S31. Crystal field energy spectrum of 5Dy representing the likely energy barrier calculated using the transition probability between each state, showing likely relaxation across the barrier from the 3rd excited state.

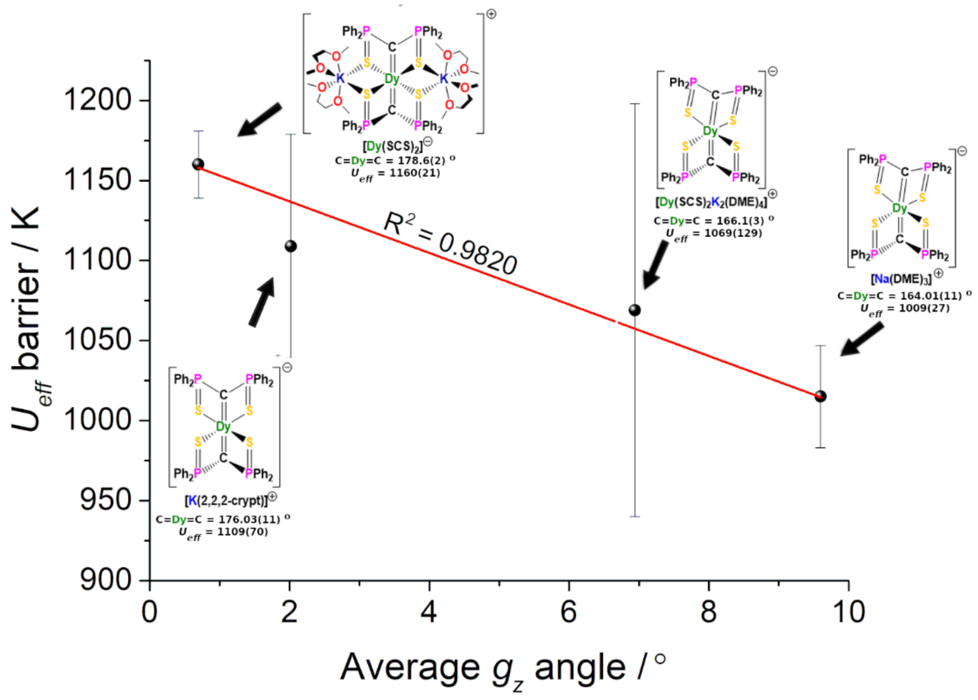

Figure S32. U_{eff} barrier as a function of the average ∠g_z-Dy=C angle.

Tables

Table S1. Experimental X-ray crystallographic details for 1Dy-5Dy, 1Y-4Y and 3Gd.

	**1Dy**	**1Y**	**2Dy**
**Formula**	$C_{68}H_{65}DyOP_4S_4$	$C_{54}H_{49}OP_4S_4Y$	$C_{116}H_{120}Dy_2K_2O_8P_8S_8$
**Fw, g mol^{-1}**	1312.82	1054.96	2549.55
**Crystal size, mm**	0.6875 x 0.2462 x 0.1786	0.132 x 0.075 x 0.06	0.2 x 0.1 x 0.1
**Crystal system**	triclinic	monoclinic	triclinic
**Space group**	P-1	P121/n1	P-1
**Collection Temperature (K)**	100(2)	150(2)	150(2)
**a, (Å)**	11.2686(6)	10.8536(6)	13.5210(2)
**b, (Å)**	12.7447(3)	22.5853(10)	20.9074(3)
**c, (Å)**	21.7296(12)	21.0093(11)	22.2017(3)
**α, (°)**	89.265(3)	90	71.6450(10)
**β, (°)**	88.319(4)	99.013(6)	79.4080(10)
**γ, (°)**	83.122(3)	90	84.3730(10)
**V, (Å^3)**	3096.8(2)	5086.5(5)	5849.94(15)
**Z**	2	4	2
**ρcalc g cm^{-3}**	1.408	1.378	1.447
**μ, mm^{-1}**	1.487	1.475	10.186
**No. of reflections measured**	22189	22004	66763
**No. of unique reflections, Rint**	13948, 0.0424	8952, 0.0874	20731, 0.0938
**No. of reflections with F2 > 2s(F2)**	10859	5840	17211
**Transmission coefficient range**	0.322-0.658	0.864-0.940	0.21935-1.00000
**R, Rwa (F2 > 2s(F2))**	0.0550, 0.1124	0.0705, 0.0968	0.0857, 0.2275
**R, Rwa (all data)**	0.0764, 0.1212	0.1213, 0.1114	0.1011, 0.2482
**Sa**	1.034	1.032	1.065
**Parameters, Restraints**	705, 0	577, 0	1305, 22
**Max.,min. difference map, e Å^{-3}**	2.073, -2.144	0.516, -0.440	6.105, -3.214

	2Y	3Dy	3Gd
**Formula**	$C_{116}H_{120}K_2O_8P_8S_8Y_2$	$C_{62}H_{70}DyNaO_6P_4S_4$	$C_{62}H_{70}GdNaO_6P_4S_4$
**Fw, g mol^{-1}**	2402.37	1348.79	1343.54
**Crystal size, mm**	0.336 x 0.136 x 0.09	0.325 x 0.187 x 0.102	0.239 x 0.109 x 0.059
**Crystal system**	triclinic	monoclinic	monoclinic
**Space group**	P-1	P21/c	P121/c1
**Collection Temperature (K)**	100(2)	150(2)	150(2)
**a, (Å)**	13.4728(4)	14.1523(4)	14.1998(4)
**b, (Å)**	20.8633(5)	30.8391(9)	30.9404(6)
**c, (Å)**	22.1735(4)	15.6028(5)	15.6211(5)
**α, (°)**	71.523(2)	90	90
**β, (°)**	79.315(2)	110.560(4)	110.508(3)
**γ, (°)**	84.386(2)	90	90
**V, (Å^3)**	5803.8(3)	6376.0(4)	6428.1(3)
**Z**	2	4	4
**ρcalc g cm^{-3}**	1.375	1.405	1.388
**μ, mm^{-1}**	1.375	1.458	1.315
**No. of reflections measured**	98260	32342	156285
**No. of unique reflections, Rint**	28623, 0.0631	13023, 0.0527	14169, 0.1779
**No. of reflections with F2 > 2s(F2)**	20852	9685	9013
**Transmission coefficient range**	0.686-1.000	0.705-1.000	0.718-1.000
**R, Rwa (F2 > 2s(F2))**	0.0682, 0.1374	0.0459, 0.0637	0.0589, 0.1265
**R, Rwa (all data)**	0.1035, 0.1486	0.0742, 0.0709	0.1113, 0.1520
**Sa**	1.064	1.024	1.023
**Parameters, Restraints**	1305, 0	709, 0	709, 0
**Max.,min. difference map, e Å^{-3}**	1.609, -0.735	0.757, -0.638	1.867, -1.417

	**3Y**	**4Dy**	**4Y**
**Formula**	$C_{62}H_{70}NaO_6P_4S_4Y$	$C_{72}H_{84}DyKN_2O_7P_4S_4$	$C_{76}H_{92}KN_2O_8P_4S_4Y$
**Fw, g mol-1**	1275.20	1543.13	1541.64
**Crystal size, mm**	0.658 x 0.443 x 0.244	0.441 x 0.159 x 0.11	0.959 x 0.732 x 0.53
**Crystal system**	monoclinic	triclinic	triclinic
**Space group**	P121/c1	P-1	P-1
**Collection Temperature (K)**	150(2)	150(2)	150(2)
**a, (Å)**	14.1550(4)	15.0677(3)	11.9072(4)
**b, (Å)**	30.9664(6)	15.8625(4)	12.5346(5)
**c, (Å)**	15.6320(5)	17.3285(3)	26.2160(11)
**α, (°)**	90	97.435(2)	81.430(3)
**β, (°)**	110.731(3)	91.979(2)	83.556(3)
**γ, (°)**	90	116.190(2)	84.318(3)
**V, (Å3)**	6408.3(3)	3665.65(15)	3831.2(3)
**Z**	4	2	2
**ρcalc g cm-3**	1.322	1.398	1.336
**μ, mm-1**	1.194	1.329	1.062
**No. of reflections measured**	31695	47182	29249
**No. of unique reflections, Rint**	14899, 0.0506	15484, 0.0401	12993, 0.0856
**No. of reflections with F2 > 2s(F2)**	9685	14161	9096
**Transmission coefficient range**	0.502-1.000	0.309-1.000	0.148-1.000
**R, Rwa (F2 > 2s(F2))**	0.0588, 0.0934	0.0377, 0.1013	0.0772, 0.2023
**R, Rwa (all data)**	0.1099, 0.1104	0.0418, 0.1030	0.1110, 0.2608
**Sa**	1.034	1.075	1.015
**Parameters, Restraints**	709, 0	815, 0	911, 480
**Max.,min. difference map, e Å-3**	0.492, -0.735	2.703, -1.205	1.506, -2.107

	5Dy
**Formula**	$C_{93}H_{116}DyKN_4O_7P_4Si_4$
**Fw, g mol-1**	1839.73
**Crystal size, mm**	0.2776 x 0.1959 x 0.1158
**Crystal system**	monoclinic
**Space group**	P121/n1
**Collection Temperature (K)**	120(2)
**a, (Å)**	13.23949(14)
**b, (Å)**	23.5137(3)
**c, (Å)**	30.0645(4)
**α, (°)**	90
**β, (°)**	97.5329(11)
**γ, (°)**	90
**V, (Å3)**	9278.6(2)
**Z**	4
**ρcalc g cm-3**	1.317
**μ, mm-1**	6.284
**No. of reflections measured**	39163
**No. of unique reflections, Rint**	18298, 0.0468
**No. of reflections with F2 > 2s(F2)**	16808
**Transmission coefficient range**	0.343-0.619
**R, Rwa (F2 > 2s(F2))**	0.0484, 0.1279
**R, Rwa (all data)**	0.0523, 0.1329
**Sa**	1.036
**Parameters, Restraints**	1040, 3
**Max.,min. difference map, e Å-3**	1.094, -1.496

Table S2. Selected bond lengths (Å) for 1Dy-5Dy, 1Y-4Y and 3Gd.

**1Dy**	**1Y**	**2Dy-anion**	**2Dy-cation**
Dy1-S1 2.8251(13)	Y1-S1 2.8245(14)	Dy1-S1 2.777(2)	Dy2-S5 2.780(2)
Dy1-S2 2.8204(12)	Y1-S2 2.8396(14)	Dy1-S2 2.771(2)	Dy2-S6 2.797(2)
Dy1-S3 2.8617(12)	Y1-S3 2.7955(15)	Dy1-S3 2.7791(19)	Dy2-S7 2.769(3)
Dy1-S4 2.7889(12)	Y1-S4 2.8643(14)	Dy1-S4 2.806(2)	Dy2-S8 2.783(2)
Dy1-O1 2.404(3)	Y1-O1 2.344(4)	Dy1-C1 2.432(7)	Dy2-C51 2.415(7)
Dy1-C1 2.325(5)	Y1-C1 2.344(5)	Dy1-C26 2.409(8)	Dy2-C76 2.390(7)
Dy1-C26 2.754(4)	Y1-C26 2.756(4)		
**2Y**	**3Dy**	**3Gd**	**3Y**
Y1-S1 2.7923(10)	Dy1-S1 2.7832(9)	Gd1-S1 2.8013(15)	Y1-S1 2.7699(9)
Y1-S2 2.7697(10)	Dy1-S2 2.7430(10)	Gd1-S2 2.7632(15)	Y1-S2 2.8126(10)
Y1-S3 2.7574(10)	Dy1-S3 2.7725(10)	Gd1-S3 2.7962(14)	Y1-S3 2.7762(9)
Y1-S4 2.7643(10)	Dy1-S4 2.8189(10)	Gd1-S4 2.8363(15)	Y1-S4 2.7366(10)
Y1-C1 2.420(3)	Dy1-C1 2.449(3)	Gd1-C1 2.507(5)	Y1-C1 2.409(3)
Y1-C26 2.445(3)	Dy1-C26 2.407(3)	Gd1-C26 2.449(5)	Y1-C26 2.464(3)
**4Dy**	**4Y**	**5Dy**	
Dy1-S1 2.8043(9)	Y1-S1 2.8874(16)	Dy1-N1 2.515(2)	
Dy1-S2 2.8246(8)	Y1-S2 2.7713(14)	Dy1-N2 2.494(2)	
Dy1-S3 2.7919(9)	Y1-S3 2.8621(17)	Dy1-N3 2.473(2)	
Dy1 -S4 2.7807(8)	Y1-S4 2.8729(15)	Dy1-N4 2.464(2)	
Dy1-C1 2.381(4)	Y1-O1 2.403(4)	Dy1-C1 2.433(3)	
Dy1-C26 2.387(3)	Y1-C1 2.589(6)	Dy1-C32 2.476(3)	
	Y1-C26 2.428(6)		

Table S3. Fitting parameters obtained from CCFit-2 using 2 processes for the AC data for 2Dy from 42 K to 63 K.

T (K)	$\chi_{S,TOT}$ (cm^3 mol^{-1})	$\Delta\chi_1$ (cm^3 mol^{-1})	τ_1 (s)	α_1	$\Delta\chi_2$ (cm^3 mol^{-1})	τ_2 (s)	α_2	Residual
42	8.68 x 10^{-2}	0.26	5.92 x 10^{-2}	0.00	0.25	5.06 x 10^{-1}	0.00	2.20 x 10^{-3}
43	8.57 x 10^{-2}	0.25	3.63 x 10^{-2}	0.00	0.24	3.61 x 10^{-1}	0.00	1.88 x 10^{-3}
44	8.39 x 10^{-2}	0.25	2.26 x 10^{-2}	0.01	0.23	2.50 x 10^{-1}	0.00	3.43 x 10^{-3}
45	7.89 x 10^{-2}	0.28	1.55 x 10^{-2}	0.09	0.20	1.75 x 10^{-1}	0.00	6.83 x 10^{-4}
46	7.53 x 10^{-2}	0.28	9.73 x 10^{-3}	0.11	0.19	1.15 x 10^{-1}	0.00	8.21 x 10^{-4}
47	7.36 x 10^{-2}	0.27	5.93 x 10^{-3}	0.11	0.19	7.18 x 10^{-2}	0.00	1.14 x 10^{-3}
48	7.10 x 10^{-2}	0.27	3.78 x 10^{-3}	0.12	0.19	4.52 x 10^{-2}	0.00	6.39 x 10^{-4}
49	6.63 x 10^{-2}	0.27	2.45 x 10^{-3}	0.13	0.18	2.90 x 10^{-2}	0.00	6.17 x 10^{-4}
50	6.16 x 10^{-2}	0.28	1.63 x 10^{-3}	0.14	0.17	1.91 x 10^{-2}	0.00	5.32 x 10^{-4}
51	5.33 x 10^{-2}	0.29	1.12 x 10^{-3}	0.18	0.15	1.25 x 10^{-2}	0.00	2.73 x 10^{-3}
52	4.80 x 10^{-2}	0.28	6.81 x 10^{-4}	0.16	0.16	7.94 x 10^{-3}	0.00	2.87 x 10^{-4}
53	3.45 x 10^{-2}	0.29	4.53 x 10^{-4}	0.18	0.15	5.23 x 10^{-3}	0.00	2.36 x 10^{-4}
54	3.04 x 10^{-2}	0.29	3.05 x 10^{-4}	0.18	0.15	3.46 x 10^{-3}	0.00	7.08 x 10^{-4}
55	2.20 x 10^{-2}	0.29	2.01 x 10^{-4}	0.15	0.16	2.31 x 10^{-3}	0.00	9.94 x 10^{-5}
56	1.16 x 10^{-2}	0.28	1.25 x 10^{-4}	0.12	0.16	1.60 x 10^{-3}	0.00	4.38 x 10^{-3}
57	1.03 x 10^{-2}	0.28	9.74 x 10^{-5}	0.09	0.16	1.12 x 10^{-3}	0.00	1.69 x 10^{-4}
58	5.39 x 10^{-16}	0.29	7.07 x 10^{-5}	0.11	0.15	7.96 x 10^{-4}	0.00	2.37 x 10^{-4}
59	1.25 x 10^{-16}	0.27	5.41 x 10^{-5}	0.08	0.16	5.54 x 10^{-4}	0.00	1.90 x 10^{-4}
60	2.09 x 10^{-16}	0.28	4.31 x 10^{-5}	0.08	0.15	4.14 x 10^{-4}	0.00	1.31 x 10^{-4}
61	1.78 x 10^{-16}	0.29	3.91 x 10^{-5}	0.09	0.13	3.20 x 10^{-4}	0.00	1.56 x 10^{-4}
62	2.82 x 10^{-16}	0.32	4.66 x 10^{-5}	0.00	0.09	3.14 x 10^{-4}	0.00	5.75 x 10^{-4}
63	1.44 x 10^{-16}	0.34	4.42 x 10^{-5}	0.00	0.06	2.96 x 10^{-4}	0.00	4.55 x 10^{-4}

Table S4. Fitting parameters obtained from CCFit-2 using a single process from for the AC data for 2Dy 26 K to 38 K. Temperatures 40 and 41 K can be fitted equally well using a single or double process, so this region cannot be trusted with either fit.

T (K)	χ_S (cm^3 mol^{-1})	χ_T (cm^3 mol^{-1})	τ (s)	α	Residual
26	0.12	1.03	3.43	0.15	4.30 x 10^{-3}
28	0.11	0.96	2.79	0.13	4.02 x 10^{-3}
30	0.11	0.87	2.21	0.12	2.46 x 10^{-3}
32	0.10	0.81	1.76	0.11	4.86 x 10^{-3}
34	0.10	0.75	1.39	0.10	2.14 x 10^{-3}
36	0.09	0.71	1.06	0.11	2.10 x 10^{-3}
38	0.09	0.67	0.70	0.13	1.91 x 10^{-3}

Table S5. Fitting of DC decay relaxation for 2Dy using equation 3.

T (K)	M_0 (emu)	M_1 (emu)	τ (s)	*β*
2	0.0207	-4.44×10^{-4}	72.62	0.938
error	-	3.69×10^{-6}	0.09	0.002
3	0.0352	-4.52×10^{-4}	108.46	0.912
error	-	7.44×10^{-6}	0.17	0.002
4	0.0399	-4.98×10^{-4}	131.62	0.880
error	-	1.06×10^{-5}	0.25	0.002
5	0.0496	-5.15×10^{-4}	149.02	0.835
error	-	1.49×10^{-5}	0.38	0.003
6	0.0549	-5.56×10^{-4}	163.89	0.794
error	-	1.80×10^{-5}	0.53	0.003
7	0.0575	-6.52×10^{-4}	180.41	0.784
error	-	1.88×10^{-5}	0.64	0.003
8	0.0635	-9.68×10^{-4}	192.46	0.781
error	-	1.95×10^{-5}	0.63	0.003
10	0.0725	-1.44×10^{-3}	202.13	0.763
error	-	2.48×10^{-5}	0.72	0.003

Table S6. Fitting parameters obtained from CCFit-2 using a single process for 3Dy from 30 K to 64 K.

T (K)	χ_S (cm^3 mol^{-1})	χ_T (cm^3 mol^{-1})	τ (s)	α	Residual
30	3.56 x 10^{-2}	0.53	2.02	0.02	6.05 x 10^{-4}
32	3.38 x 10^{-2}	0.49	1.58	0.02	3.40 x 10^{-4}
34	3.22 x 10^{-2}	0.46	1.18	0.01	3.88 x 10^{-4}
36	3.09 x 10^{-2}	0.43	7.35 x 10^{-1}	0.01	3.81 x 10^{-4}
38	2.97 x 10^{-2}	0.41	3.40 x 10^{-1}	0.01	4.48 x 10^{-4}
40	2.89 x 10^{-2}	0.39	1.28 x 10^{-1}	0.02	2.89 x 10^{-4}
41	2.78 x 10^{-2}	0.38	7.52 x 10^{-2}	0.02	1.74 x 10^{-4}
42	2.77 x 10^{-2}	0.37	4.40 x 10^{-2}	0.02	5.40 x 10^{-4}
43	2.69 x 10^{-2}	0.34	2.48 x 10^{-2}	0.00	4.82 x 10^{-2}
44	2.72 x 10^{-2}	0.35	1.56 x 10^{-2}	0.00	3.89 x 10^{-4}
45	2.78 x 10^{-2}	0.34	9.54 x 10^{-3}	0.00	8.58 x 10^{-4}
46	2.57 x 10^{-2}	0.34	5.85 x 10^{-3}	0.00	6.38 x 10^{-4}
47	2.60 x 10^{-2}	0.33	3.68 x 10^{-3}	0.00	3.42 x 10^{-4}
48	2.57 x 10^{-2}	0.32	2.36 x 10^{-3}	0.00	3.99 x 10^{-4}
49	2.50 x 10^{-2}	0.32	1.53 x 10^{-3}	0.00	3.12 x 10^{-4}
50	2.40 x 10^{-2}	0.31	1.01 x 10^{-3}	0.00	2.36 x 10^{-4}
51	2.20 x 10^{-2}	0.31	6.76 x 10^{-4}	0.00	1.50 x 10^{-4}
52	1.91 x 10^{-2}	0.30	4.54 x 10^{-4}	0.00	2.72 x 10^{-4}
53	1.95 x 10^{-2}	0.29	3.15 x 10^{-4}	0.00	2.03 x 10^{-4}
54	1.70 x 10^{-2}	0.29	2.22 x 10^{-4}	0.00	2.20 x 10^{-3}
55	1.56 x 10^{-2}	0.28	1.56 x 10^{-4}	0.00	1.25 x 10^{-4}
56	1.38 x 10^{-2}	0.28	1.13 x 10^{-4}	0.00	1.12 x 10^{-4}
57	2.69 x 10^{-2}	0.27	8.91 x 10^{-5}	0.00	4.03 x 10^{-4}
58	5.82 x 10^{-3}	0.27	6.00 x 10^{-5}	0.00	3.41 x 10^{-4}
59	1.00 x 10^{-2}	0.27	4.52 x 10^{-5}	0.00	2.48 x 10^{-4}
60	8.57 x 10^{-3}	0.26	3.68 x 10^{-5}	0.00	6.92 x 10^{-5}
61	2.01 x 10^{-9}	0.26	2.74 x 10^{-5}	0.00	9.67 x 10^{-5}
62	2.97 x 10^{-9}	0.25	2.16 x 10^{-5}	0.00	6.82 x 10^{-5}
63	4.28 x 10^{-9}	0.25	1.88 x 10^{-5}	0.00	7.03 x 10^{-4}
64	6.33 x 10^{-9}	2.46 x 10^{-1}	1.45 x 10^{-5}	0.00	5.88 x 10^{-4}

Table S7. Fitting parameters obtained from CCFit-2 using a single process for 4Dy from 30 K to 70 K.

T (K)	χ_S (cm^3 mol^{-1})	χ_T (cm^3 mol^{-1})	τ (s)	α	Residual
30	2.64 x 10^{-2}	0.66	7.28	0.16	6.07 x 10^{-4}
32	2.60 x 10^{-2}	0.57	5.19	0.14	7.42 x 10^{-4}
34	2.47 x 10^{-2}	0.50	3.74	0.13	5.35 x 10^{-4}
36	2.48 x 10^{-2}	0.44	2.56	0.12	6.13 x 10^{-4}
38	2.44 x 10^{-2}	0.40	1.71	0.11	7.04 x 10^{-4}
40	2.48 x 10^{-2}	0.36	9.24 x 10^{-1}	0.10	9.56 x 10^{-4}
42	2.48 x 10^{-2}	0.34	4.09 x 10^{-1}	0.09	8.45 x 10^{-4}
44	2.47 x 10^{-2}	0.32	1.59 x 10^{-1}	0.08	7.61 x 10^{-4}
46	2.44 x 10^{-2}	0.30	5.88 x 10^{-2}	0.08	5.85 x 10^{-4}
48	2.41 x 10^{-2}	0.29	2.25 x 10^{-2}	0.07	5.73 x 10^{-4}
50	2.37 x 10^{-2}	0.28	9.03 x 10^{-3}	0.07	6.27 x 10^{-4}
52	2.25 x 10^{-2}	0.27	3.83 x 10^{-3}	0.07	4.74 x 10^{-4}
54	2.15 x 10^{-2}	0.26	1.72 x 10^{-3}	0.06	3.72 x 10^{-4}
56	1.92 x 10^{-2}	0.25	8.13 x 10^{-4}	0.06	4.08 x 10^{-4}
58	1.72 x 10^{-2}	0.24	4.02 x 10^{-4}	0.05	3.04 x 10^{-4}
60	1.33 x 10^{-2}	0.23	2.10 x 10^{-4}	0.05	2.09 x 10^{-4}
62	9.66 x 10^{-3}	0.22	1.17 x 10^{-4}	0.03	1.50 x 10^{-4}
64	1.85 x 10^{-2}	0.22	7.29 x 10^{-5}	0.01	3.93 x 10^{-4}
66	9.78 x 10^{-9}	0.21	4.01 x 10^{-5}	0.02	1.10 x 10^{-4}
68	2.08 x 10^{-8}	0.20	2.77 x 10^{-5}	0.00	1.36 x 10^{-4}
70	3.19 x 10^{-8}	0.20	1.95 x 10^{-5}	0.00	1.87 x 10^{-4}

Table S8. Fitting of DC decay relaxation for 3Dy using equation 3.

T (K)	M_0 (emu)	M_1 (emu)	τ (s)	b
2	0.0217	-4.36 x 10^{-4}	65.19	0.951
error	-	5.14 x 10^{-6}	0.14	0.003
3	0.0393	-4.88 x 10^{-4}	95.46	0.921
error	-	7.67 x 10^{-6}	0.19	0.003
4	0.0488	-5.32 x 10^{-4}	112.49	0.904
error	-	9.66 x 10^{-6}	0.24	0.003
5	0.0587	-5.67 x 10^{-4}	128.24	0.878
error	-	9.60 x 10^{-6}	0.27	0.002
6	0.0687	-6.80 x 10^{-4}	146.31	0.854
error	-	9.00 x 10^{-6}	0.29	0.002
7	0.0806	-8.09 x 10^{-4}	169.84	0.827
error	-	8.75 x 10^{-6}	0.30	0.002
8	0.1020	-9.96 x 10^{-4}	202.73	0.799
error	-	1.06 x 10^{-5}	0.35	0.002
10	0.1402	-1.45 x 10^{-3}	253.93	0.734
error	-	2.93 x 10^{-5}	0.67	0.002

Table S9. Fitting of DC decay relaxation for 4Dy using equation 3.

T (K)	M_0 (emu)	M_1 (emu)	τ (s)	b
2	0.0134	-1.98 x 10^{-4}	89.90	1.245
error	-	8.12 x 10^{-5}	2.61	0.063
3	0.0338	-2.02 x 10^{-4}	131.02	1.118
error	-	7.19 x 10^{-5}	1.53	0.021
4	0.0559	-2.34 x 10^{-4}	195.17	1.012
error	-	5.69 x 10^{-5}	1.21	0.009
5	0.0586	-2.91 x 10^{-4}	247.69	0.958
error	-	4.37 x 10^{-5}	1.13	0.006
6	0.0698	-3.00 x 10^{-4}	297.31	0.887
error	-	3.67 x 10^{-5}	1.12	0.004
7	0.0784	-3.03 x 10^{-4}	361.69	0.818
error	-	3.23 x 10^{-5}	1.25	0.003
8	0.0867	-1.18 x 10^{-4}	434.74	0.754
error	-	3.80 x 10^{-5}	1.59	0.003
10	0.0937	5.22 x 10^{-4}	553.71	0.694
error	-	6.25 x 10^{-5}	2.33	0.003
12	0.1100	0.00159	635.91	0.619
error	-	8.45 x 10^{-5}	3.21	0.003

Table S10. Fitting parameters obtained from CCFit-2 using a single process for 5Dy from 22 K to 50 K.

T (K)	χ_S (cm^3 mol^{-1})	χ_T (cm^3 mol^{-1})	τ (s)	α	Residual
22	2.75 x 10^{-2}	5.70 x 10^{-1}	1.61	5.30 x 10^{-2}	5.36 x 10^{-4}
23	2.69 x 10^{-2}	5.52 x 10^{-1}	1.36	5.16 x 10^{-2}	5.55 x 10^{-4}
24	2.66 x 10^{-2}	5.27 x 10^{-1}	1.10	4.83 x 10^{-2}	5.30 x 10^{-4}
25	2.61 x 10^{-2}	5.04 x 10^{-1}	8.73 x 10^{-1}	4.49 x 10^{-2}	5.99 x 10^{-4}
26	2.49 x 10^{-2}	4.83 x 10^{-1}	6.80 x 10^{-1}	4.23 x 10^{-2}	5.40 x 10^{-4}
27	2.51 x 10^{-2}	4.60 x 10^{-1}	5.04 x 10^{-1}	2.71 x 10^{-2}	1.12 x 10^{-3}
28	2.49 x 10^{-2}	4.46 x 10^{-1}	3.66 x 10^{-1}	3.99 x 10^{-2}	5.40 x 10^{-4}
29	2.39 x 10^{-2}	4.30 x 10^{-1}	2.42 x 10^{-1}	3.95 x 10^{-2}	7.08 x 10^{-4}
30	2.31 x 10^{-2}	4.15 x 10^{-1}	1.51 x 10^{-1}	4.40 x 10^{-2}	3.50 x 10^{-4}
31	2.35 x 10^{-2}	4.03 x 10^{-1}	8.73 x 10^{-2}	4.57 x 10^{-2}	3.50 x 10^{-4}
32	2.18 x 10^{-2}	3.89 x 10^{-1}	4.79 x 10^{-2}	4.69 x 10^{-2}	3.48 x 10^{-4}
33	2.27 x 10^{-2}	3.78 x 10^{-1}	2.59 x 10^{-2}	4.42 x 10^{-2}	5.23 x 10^{-4}
34	2.04 x 10^{-2}	3.67 x 10^{-1}	1.40 x 10^{-2}	4.98 x 10^{-2}	5.53 x 10^{-4}
35	2.25 x 10^{-2}	3.57 x 10^{-1}	7.64 x 10^{-3}	4.47 x 10^{-2}	2.61 x 10^{-4}
36	1.92 x 10^{-2}	3.46 x 10^{-1}	4.23 x 10^{-3}	4.61 x 10^{-2}	2.83 x 10^{-4}
37	1.77 x 10^{-2}	3.38 x 10^{-1}	2.36 x 10^{-3}	5.16 x 10^{-2}	5.34 x 10^{-4}
38	1.74 x 10^{-2}	3.29 x 10^{-1}	1.38 x 10^{-3}	4.47 x 10^{-2}	3.69 x 10^{-4}
39	1.40 x 10^{-2}	3.21 x 10^{-1}	8.09 x 10^{-4}	4.53 x 10^{-2}	2.50 x 10^{-4}

40	1.43×10^{-2}	3.13×10^{-1}	4.99×10^{-4}	4.36×10^{-2}	1.65×10^{-4}
41	1.07×10^{-2}	3.06×10^{-1}	3.05×10^{-4}	3.82×10^{-2}	1.72×10^{-4}
42	6.05×10^{-3}	2.98×10^{-1}	1.95×10^{-4}	3.08×10^{-2}	1.09×10^{-4}
43	1.41×10^{-16}	2.92×10^{-1}	1.25×10^{-4}	2.64×10^{-2}	9.07×10^{-5}
44	2.24×10^{-16}	2.84×10^{-1}	8.57×10^{-5}	1.24×10^{-2}	5.68×10^{-5}
45	2.93×10^{-16}	2.80×10^{-1}	5.83×10^{-5}	1.52×10^{-2}	8.53×10^{-5}
46	3.77×10^{-16}	2.72×10^{-1}	4.31×10^{-5}	8.53×10^{-15}	1.72×10^{-4}
47	5.62×10^{-16}	2.69×10^{-1}	3.01×10^{-5}	9.72×10^{-15}	7.01×10^{-5}
48	7.60×10^{-16}	2.62×10^{-1}	2.24×10^{-5}	8.87×10^{-15}	5.53×10^{-4}
49	1.08×10^{-15}	2.59×10^{-1}	1.63×10^{-5}	8.31×10^{-15}	9.60×10^{-5}
50	1.58×10^{-15}	2.53×10^{-1}	1.52×10^{-5}	1.08×10^{-14}	4.06×10^{-3}

Table S11. Fitting of DC decay relaxation for 5Dy using equation 3.

T (K)	M_0 (emu)	M_1 (emu)	τ (s)	b
2	0.0011	-3.21×10^{-4}	32.46	0.992
error	-	1.03×10^{-6}	0.13	0.006
3	0.0097	-3.92×10^{-4}	43.66	0.947
error	-	1.59×10^{-6}	0.05	0.002
4	0.0114	-4.32×10^{-4}	51.11	0.956
error	-	2.58×10^{-6}	0.07	0.002
5	0.0189	-4.81×10^{-4}	56.59	0.951
error	-	4.59×10^{-6}	0.09	0.002
6	0.0232	-5.89×10^{-4}	65.09	0.950
error	-	6.80×10^{-6}	0.12	0.002
7	0.0324	-8.01×10^{-4}	87.42	0.972
error	-	5.07×10^{-5}	0.82	0.013
8	0.0527	-1.09×10^{-3}	96.62	0.917
error	-	5.32×10^{-5}	0.55	0.007
10	0.0835	-2.39×10^{-3}	148.26	0.787
error	-	1.40×10^{-4}	1.26	0.008

Table S12. Wavefunction decomposition calculated in the basis of the principal axis of the ground doublet for 2Dy-anion. The ground g_z orientation is defined by the principal g-value of the ground doublet, with the angle to subsequent g_z calculated relative to the ground state principal axis.

**Energy (cm^{-1})**	**Energy (K)**	g_x	g_y	g_z	**Angle (°)**	**Wavefunction**
0.0	0.0	0.00	0.00	19.88	--	99.9%|±15/2⟩
296.6	426.7	0.00	0.00	17.09	2.8	98.9%|±13/2⟩ 0.6%|±11/2⟩ 0.1%|±9/2⟩ 0.2%|±7/2⟩ 0.1%|±5/2⟩
572.8	824.2	0.03	0.03	14.14	5.8	0.5%|±13/2⟩ 95.5%|±11/2⟩ 1.4%|±7/2⟩ 1.5%|±5/2⟩ 0.9%|±3/2⟩ 0.2%|±1/2⟩
717.6	1032.5	0.60	1.15	14.77	61.2	0.3%|±13/2⟩ 1.5%|±11/2⟩ 35.2%|±9/2⟩ 9%|±7/2⟩ 14.6%|±5/2⟩ 22.2%|±3/2⟩ 17.3%|±1/2⟩
776.9	1117.8	2.14	2.40	14.48	70.1	0.6%|±11/2⟩ 32.7%|±9/2⟩ 4.7%|±7/2⟩ 11.6%|±5/2⟩ 10.5%|±3/2⟩ 39.8%|±1/2⟩
815.3	1173.1	1.99	3.86	6.67	49.9	0.1%|±13/2⟩ 0.9%|±11/2⟩ 27%|±9/2⟩ 15.5%|±7/2⟩ 10.1%|±5/2⟩ 22.4%|±3/2⟩ 23.9%|±1/2⟩
873.1	1256.2	3.03	5.75	11.77	69.3	0.1%|±13/2⟩ 0.8%|±11/2⟩ 3.3%|±9/2⟩ 46.4%|±7/2⟩ 16.3%|±5/2⟩ 22.3%|±3/2⟩ 10.9%|±1/2⟩
946.1	1361.2	0.34	0.85	17.89	70.3	0.1%|±11/2⟩ 1.6%|±9/2⟩ 22.8%|±7/2⟩ 45.8%|±5/2⟩ 21.7%|±3/2⟩ 8%|±1/2⟩

Table S13. Wavefunction decomposition calculated in the basis of the principal axis of the ground doublet for 2Dy-cation. The ground g_z orientation is defined by the principal g-value of the ground doublet, with the angle to subsequent g_z calculated relative to the ground state principal axis.

Energy (cm^{-1})	Energy (K)	g_x	g_y	g_z	Angle (°)	Wavefunction
0.0	0.0	0.00	0.00	19.89	--	100%|±15/2⟩
325.8	468.8	0.00	0.00	17.09	0.2	99.9%|±13/2⟩ 0.1%|±5/2⟩
644.2	926.9	0.00	0.02	14.23	0.4	99%|±11/2⟩ 1%|±3/2⟩
831.2	1196.0	3.93	7.37	10.48	89.5	28%|±9/2⟩ 2.7%|±7/2⟩ 0.2%|±5/2⟩ 1%|±3/2⟩ 68.2%|±1/2⟩
884.2	1272.2	2.73	3.76	7.79	79.4	0.5%|±11/2⟩ 30.6%|±9/2⟩ 0.8%|±7/2⟩ 7.4%|±5/2⟩ 53.2%|±3/2⟩ 7.3%|±1/2⟩
892.2	1283.7	1.60	3.13	9.97	67.4	0.3%|±11/2⟩ 40.7%|±9/2⟩ 2.8%|±7/2⟩ 7.7%|±5/2⟩ 30.1%|±3/2⟩ 18.2%|±1/2⟩
959.5	1380.5	0.20	3.01	11.97	79.4	0.1%|±13/2⟩ 0.1%|±11/2⟩ 0.2%|±9/2⟩ 17.5%|±7/2⟩ 70.3%|±5/2⟩ 11.1%|±3/2⟩ 0.7%|±1/2⟩
974.3	1401.9	2.94	7.79	9.11	89.8	0.5%|±9/2⟩ 76.1%|±7/2⟩ 14.2%|±5/2⟩ 3.6%|±3/2⟩ 5.6%|±1/2⟩

Table S14. Wavefunction decomposition calculated in the basis of the principal axis of the ground doublet for 3Dy. The ground g_z orientation is defined by the principal g-value of the ground doublet, with the angle to subsequent g_z calculated relative to the ground state principal axis.

Energy (cm^{-1})	Energy (K)	g_x	g_y	g_z	Angle (°)	Wavefunction
0.0	0.0	0.00	0.00	19.88	--	99.8%|±15/2⟩ 0.1%|±13/2⟩
291.9	419.9	0.00	0.00	17.09	3.2	0.1%|±15/2⟩ 98.7%|±13/2⟩ 0.9%|±11/2⟩ 0.1%|±9/2⟩ 0.2%|±7/2⟩ 0.1%|±5/2⟩
554.4	797.6	0.04	0.05	14.15	6.8	0.7%|±13/2⟩ 95%|±11/2⟩ 0.1%|±9/2⟩ 1.1%|±7/2⟩ 2%|±5/2⟩ 1.1%|±3/2⟩ 0.1%|±1/2⟩
694.3	998.9	0.30	0.66	14.40	55.2	0.4%|±13/2⟩ 1.5%|±11/2⟩ 45.9%|±9/2⟩ 11.3%|±7/2⟩ 13.2%|±5/2⟩ 16.8%|±3/2⟩ 11%|±1/2⟩
745.2	1072.2	1.70	2.26	16.80	68.7	0.1%|±13/2⟩ 1.6%|±11/2⟩ 21.9%|±9/2⟩ 14.6%|±7/2⟩ 20.4%|±5/2⟩ 24.2%|±3/2⟩ 17.1%|±1/2⟩
789.4	1135.8	6.25	4.76	0.59	79.9	0.6%|±11/2⟩ 27.7%|±9/2⟩ 10.6%|±7/2⟩ 7.8%|±5/2⟩ 3.8%|±3/2⟩ 49.5%|±1/2⟩
858.3	1234.9	3.03	4.22	9.15	55.5	0.4%|±11/2⟩ 4.3%|±9/2⟩ 43.7%|±7/2⟩ 1.9%|±5/2⟩ 30.5%|±3/2⟩ 19.1%|±1/2⟩
921.2	1325.4	0.46	1.80	16.28	67.0	0.1%|±11/2⟩ 18.3%|±7/2⟩ 54.7%|±5/2⟩ 23.7%|±3/2⟩ 3.2%|±1/2⟩

Table S15. Wavefunction decomposition calculated in the basis of the principal axis of the ground doublet for 4Dy. The ground g_z orientation is defined by the principal g-value of the ground doublet, with the angle to subsequent g_z calculated relative to the ground state principal axis.

Energy (cm^{-1})	Energy (K)	g_x	g_y	g_z	Angle (°)	Wavefunction
0.0	0.0	0.00	0.00	19.89	--	100%|±15/2⟩
327.6	471.3	0.00	0.00	17.09	0.8	99.9%|±13/2⟩ 0.1%|±5/2⟩
57.9	946.6	0.01	0.02	14.25	0.6	99.3%|±11/2⟩ 0.6%|±3/2⟩
861.1	1238.9	3.20	4.95	12.74	88.4	0.1%|±11/2⟩ 27%|±9/2⟩ 2.1%|±7/2⟩ 1.8%|±5/2⟩ 8.2%|±3/2⟩ 60.9%|±1/2⟩
908.1	1306.6	2.83	4.82	6.89	13.5	0.2%|±11/2⟩ 63%|±9/2⟩ 2.1%|±7/2⟩ 2.4%|±5/2⟩ 18.8%|±3/2⟩ 13.5%|±1/2⟩
930.2	1338.3	0.11	1.59	14.49	83.4	0.4%|±11/2⟩ 8%|±9/2⟩ 3.4%|±7/2⟩ 18%|±5/2⟩ 54.5%|±3/2⟩ 15.7%|±1/2⟩
986.3	1419.0	9.91	7.70	2.96	29.0	0.1%|±11/2⟩ 0.7%|±9/2⟩ 48.5%|±7/2⟩ 40%|±5/2⟩ 6.2%|±3/2⟩ 4.5%|±1/2⟩
1031.9	1484.7	0.70	1.64	16.74	68.5	1.3%|±9/2⟩ 43.8%|±7/2⟩ 37.7%|±5/2⟩ 11.7%|±3/2⟩ 5.4%|±1/2⟩

Table 16. Wavefunction decomposition calculated in the basis of the principal axis of the ground doublet for 5Dy. The ground g_z orientation is defined by the principal g-value of the ground doublet, with the angle to subsequent g_z calculated relative to the ground state principal axis.

Energy (cm^{-1})	Energy (K)	g_x	g_y	g_z	Angle (°)	Wavefunction
0.0	0.0	0.00	0.00	19.88	--	99.6%|±15/2⟩ 0.3%|±13/2⟩ 0.1%|±11/2⟩
170.5	245.3	0.00	0.00	17.18	4.6	0.3%|±15/2⟩ 99.4%|±13/2⟩ 0.1%|±11/2⟩ 0.2%|±9/2⟩
398.0	572.6	0.31	0.50	14.04	1.6	0.1%|±15/2⟩ 0.1%|±13/2⟩ 96%|±11/2⟩ 0.8%|±7/2⟩ 0.6%|±5/2⟩ 1.9%|±3/2⟩ 0.5%|±1/2⟩
481.5	692.8	1.06	2.86	16.63	82.8	0.1%|±13/2⟩ 2%|±11/2⟩ 10%|±9/2⟩ 4.8%|±7/2⟩ 7.8%|±5/2⟩ 16.9%|±3/2⟩ 58.4%|±1/2⟩
551.1	792.9	0.41	1.16	15.98	79.5	0.6%|±11/2⟩ 8.6%|±9/2⟩ 8.3%|±7/2⟩ 17.8%|±5/2⟩ 47.3%|±3/2⟩ 17.4%|±1/2⟩
582.7	838.4	0.87	4.54	8.87	20.9	0.1%|±13/2⟩ 0.3%|±11/2⟩ 69.6%|±9/2⟩ 1.1%|±7/2⟩ 7.7%|±5/2⟩ 9.6%|±3/2⟩ 11.6%|±1/2⟩
647.1	931.1	10.51	6.19	0.86	12.9	0.3%|±11/2⟩ 7.7%|±9/2⟩ 45.1%|±7/2⟩ 34.6%|±5/2⟩ 9.2%|±3/2⟩ 3%|±1/2⟩
693.1	997.3	1.33	3.19	16.64	81.5	0.5%|±11/2⟩ 4%|±9/2⟩ 39.8%|±7/2⟩ 31.5%|±5/2⟩ 15%|±3/2⟩ 9.1%|±1/2⟩

Table S17. Crystal field parameters (including operator equivalent factors) in the basis of the principal axis of the ground doublet state for all analogues, given in cm^{-1}.

	k	q	2Dy anion	2Dy cation	3Dy	4Dy	5Dy
**B**	**2**	**-2**	-6.20×10^{-1}	-5.48×10^{-2}	-1.61×10^{-1}	7.58×10^{-2}	-9.03×10^{-2}
**B**	**2**	**-1**	-7.52×10^{-1}	7.44×10^{-2}	6.57×10^{-1}	-6.91×10^{-2}	3.65×10^{-1}
**B**	**2**	**0**	-4.95	-5.38	-4.83	-5.65	-3.56
**B**	**2**	**1**	1.49×10^{-1}	-2.38×10^{-2}	9.45×10^{-1}	4.23×10^{-1}	3.77×10^{-1}
**B**	**2**	**2**	7.09×10^{-1}	1.54×10^{-1}	1.28×10^{-1}	5.64×10^{-1}	9.53×10^{-1}
**B**	**4**	**-4**	-1.00×10^{-2}	1.87×10^{-3}	5.76×10^{-3}	1.42×10^{-2}	-2.00×10^{-2}
**B**	**4**	**-3**	1.50×10^{-2}	-4.76×10^{-3}	-3.99×10^{-2}	-7.58×10^{-3}	8.67×10^{-3}
**B**	**4**	**-2**	3.25×10^{-3}	4.16×10^{-4}	3.15×10^{-4}	-4.27×10^{-4}	1.51×10^{-4}
**B**	**4**	**-1**	2.04×10^{-2}	-1.72×10^{-3}	-2.09×10^{-2}	6.65×10^{-5}	-5.20×10^{-3}
**B**	**4**	**0**	-8.43×10^{-3}	-1.04×10^{-2}	-7.71×10^{-3}	-1.03×10^{-2}	-6.96×10^{-3}
**B**	**4**	**1**	-5.09×10^{-3}	-4.63×10^{-4}	-1.47×10^{-2}	-6.04×10^{-3}	-1.75×10^{-3}
**B**	**4**	**2**	2.95×10^{-3}	-7.84×10^{-4}	4.82×10^{-3}	-2.03×10^{-3}	4.49×10^{-4}
**B**	**4**	**3**	-3.34×10^{-2}	-3.09×10^{-3}	-5.06×10^{-3}	2.86×10^{-4}	1.57×10^{-2}
**B**	**4**	**4**	-4.15×10^{-3}	1.60×10^{-2}	-7.97×10^{-3}	-4.31×10^{-4}	-1.94×10^{-3}
**B**	**6**	**-6**	-1.09×10^{-5}	2.10×10^{-7}	1.67×10^{-5}	1.73×10^{-5}	-1.29×10^{-5}
**B**	**6**	**-5**	-3.85×10^{-5}	6.81×10^{-5}	1.92×10^{-5}	2.13×10^{-6}	5.83×10^{-5}
**B**	**6**	**-4**	-1.87×10^{-5}	2.13×10^{-6}	2.87×10^{-5}	2.93×10^{-5}	6.57×10^{-5}
**B**	**6**	**-3**	-3.71×10^{-5}	-4.36×10^{-5}	3.94×10^{-6}	1.19×10^{-5}	5.50×10^{-7}
**B**	**6**	**-2**	-2.20×10^{-5}	-2.95×10^{-6}	7.50×10^{-5}	1.18×10^{-5}	-5.90×10^{-6}
**B**	**6**	**-1**	-2.47×10^{-4}	8.02×10^{-6}	2.46×10^{-4}	-6.11×10^{-6}	1.16×10^{-4}
**B**	**6**	**0**	3.81×10^{-5}	5.26×10^{-5}	3.14×10^{-5}	5.60×10^{-5}	5.38×10^{-5}
**B**	**6**	**1**	6.81×10^{-5}	1.32×10^{-6}	1.62×10^{-4}	7.64×10^{-5}	4.41×10^{-5}
**B**	**6**	**2**	-7.44×10^{-5}	1.66×10^{-5}	-5.83×10^{-5}	1.57×10^{-5}	-2.34×10^{-6}
**B**	**6**	**3**	-4.44×10^{-5}	2.37×10^{-6}	-1.37×10^{-5}	-5.02×10^{-5}	-4.14×10^{-5}
**B**	**6**	**4**	-4.34×10^{-5}	4.28×10^{-5}	-2.51×10^{-5}	-7.53×10^{-6}	3.51×10^{-5}
**B**	**6**	**5**	5.94×10^{-5}	-1.07×10^{-5}	6.75×10^{-5}	4.29×10^{-5}	-5.39×10^{-5}
**B**	**6**	**6**	-5.01×10^{-6}	9.94×10^{-6}	-1.63×10^{-6}	-7.26×10^{-6}	-5.01×10^{-6}

Table S18. Crystal field parameters (including operator equivalent factors) in the basis of the principal axis of the ground doublet state for 3Gd, given in cm^{-1}.

	k	q	CFP		k	q	CFP
**B**	2	-2	8.93 X 10^{-3}	**B**	6	-6	-1.15 X 10^{-8}
**B**	2	-1	2.82 X 10^{-2}	**B**	6	-5	5.52 X 10^{-8}
**B**	2	0	-2.60 X 10^{-2}	**B**	6	-4	2.23 X 10^{-8}
**B**	2	1	-1.00 X 10^{-1}	**B**	6	-3	1.50 X 10^{-8}
**B**	2	2	-1.04 X 10^{-2}	**B**	6	-2	1.71 X 10^{-9}
**B**	4	-4	-1.77 X 10^{-5}	**B**	6	-1	-1.58 X 10^{-8}
**B**	4	-3	-2.03 X 10^{-5}	**B**	6	0	6.03 X 10^{-9}
**B**	4	-2	-2.75 X 10^{-6}	**B**	6	1	2.15 X 10^{-8}
**B**	4	-1	-3.40 X 10^{-6}	**B**	6	2	1.33 X 10^{-8}
**B**	4	0	-3.46 X 10^{-6}	**B**	6	3	-3.82 X 10^{-8}
**B**	4	1	8.96 X 10^{-6}	**B**	6	4	-4.41 X 10^{-8}
**B**	4	2	2.93 X 10^{-5}	**B**	6	5	-3.36 X 10^{-8}
**B**	4	3	-1.47 X 10^{-5}	**B**	6	6	2.24 X 10^{-8}
**B**	4	4	2.97 X 10^{-5}				

Table S19. Angles formed by principal quantisation axes from CASSCF-SO calculations with the bis-carbene C centres, showing a preference to point towards one of the C^{2-} centres in the SCS systems, compared to finding an average point in the BIPM system where the two C=Dy bonds are similar in length.

		g_z angle (°)
**2Dy-anion**	C1=Dy-g_z	1.832
	C26=Dy- g_z	12.05
**2Dy-cation**	C51=Dy- g_z	0.84
	C76=Dy- g_z	0.552
**3Dy**	C1=Dy- g_z	5.786
	C26=Dy- g_z	13.409
**4Dy**	C1=Dy- g_z	3.495
	C26=Dy- g_z	0.542
**5Dy**	C1=Dy- g_z	4.225
	C32=Dy- g_z	3.92

Table S20. Wavefunction decomposition calculated in the basis of the principal axis of the ground doublet for 2Dy-cation with the {K(DME)$_2$}$^+$ units removed. The ground g_z orientation is defined by the principal g-value of the ground doublet, with the angle to subsequent g_z calculated relative to the ground state principal axis.

**Energy (cm^{-1})**	**Energy (K)**	g_x	g_y	g_z	**Angle (°)**	**Wavefunction**
0	0	0.00	0.00	19.88	--	100%|±15/2⟩
305	439	0.00	0.00	17.09	0.25	99.9%|±13/2⟩ 0.1%|±3/2⟩
609	876	0.04	0.06	14.21	0.31	98.7%|±11/2⟩, 1.2%|±13/2⟩
764	1100	2.31	6.01	13.43	89.59	0.1%|±11/2⟩, 13.5%|±9/2⟩, 2.1%|±7/2⟩, 0.7%|±5/2⟩, 4.4%|±3/2⟩, 79.2%|±1/2⟩
820	1180	2.39	2.77	9.07	89.29	0.9%|±11/2⟩, 5.4%|±9/2⟩, 2.7%|±7/2⟩, 11.3%|±5/2⟩, 77.5%|±3/2⟩, 2.2%|±1/2⟩
834	1200	0.38	1.68	9.70	11.00	0.1%|±11/2⟩, 78.0%|±9/2⟩, 1.0%|±7/2⟩, 5.1%|±5/2⟩, 4.2%|±3/2⟩, 11.6%|±1/2⟩
896	1290	2.69	4.24	10.09	86.51	2.1%|±9/2⟩, 21.6%|±7/2⟩, 69.9%|±5/2⟩, 5.7%|±3/2⟩, 0.7%|±1/2⟩
920	1324	3.17	6.02	11.57	87.55	0.1%|±11/2⟩, 1.0%|±9/2⟩, 72.5%|±7/2⟩, 12.9%|±5/2⟩, 7.0%|±3/2⟩, 6.3%|±1/2⟩

References

1. K. Izod, S. T. Liddle and W. Clegg, *Inorg. Chem.*, 2004, **43**, 214.
2. L. Lochmann and J. Trekoval, *J. Organomet. Chem.*, 1987, **326**, 1.
3. T. Cantat, F. Jaroschik, F. Nief, L. Ricard, N. Mézailles and P. Le Floch, *Chem. Commun.*, 2005, **41**, 5178.
4. F. Aquilante, J. Autschbach, R. K. Carlson, L. F. Chibotaru, M. G. Delcey, L. De Vico, I. Fdez. Galván, N. Ferré, L. M. Frutos, L. Gagliardi, M. Garavelli, A. Giussani, C. E. Hoyer, G. Li Manni, H. Lischka, D. Ma, P. Å. Malmqvist, T. Müller, A. Nenov, M. Olivucci, T. B. Pedersen, D. Peng, F. Plasser, B. Pritchard, M. Reiher, I. Rivalta, I. Schapiro, J. Segarra-Martí, M. Stenrup, D. G. Truhlar, L. Ungur, A. Valentini, S. Vancoillie, V. Veryazov, V. P. Vysotskiy, O. Weingart, F. Zapata and R. Lindh, J. Comput. Chem., 2016, **37**, 506.
5. B. O. Roos, R. Lindh, P.-Å. Malmqvist, V. Veryazov and P.-O. Widmark, J. Phys. Chem. A, 2008, **112**, 11431.
6. B. O. Roos, R. Lindh, P. Å. Malmqvist, V. Veryazov and P. O. Widmark, J. Phys. Chem. A, 2004, **108**, 2851.
7. G. M. Sheldrick, *Acta Cryst. Sect. A,* 2015, **71**, 3.
8. L. Palatinus, G. Chapuis, *J. Appl. Cryst.,* 2007, **40**, 786.
9. Oxford Diffraction /Agilent Technologies UK Ltd, Yarnton, E.
10. G. M. Sheldrick, *Acta Cryst. Sect. C, Struct. Chem.,* 2015, **71**, 3.
11. O. V. Dolomanov, L. J. Bourhis, R. J. Gildea, J. A. K. Howard, H. Puschmann, *J. Appl. Cryst.,* 2009, **42**, 339.
12. L. J. Farrugia, *J. Appl. Cryst.,* 2012, **45**, 849.
13. Persistence of Vision (TM) Raytracer, Persistence of Vision Pty. Ltd., Williamstown, Victoria, Australia.
